# Influence of frailty and cognitive decline on dual task performance in older adults: an analytical cross-sectional study*

**DOI:** 10.1590/1518-8345.7159.4485

**Published:** 2025-02-17

**Authors:** Francine Golghetto Casemiro, Lucas Pelegrini Nogueira de Carvalho, Fernanda de Brito Matiello, Marcela Cristina Resende, Rosalina Aparecida Partezani Rodrigues

**Affiliations:** 1 Universidade de São Paulo, Escola de Enfermagem de Ribeirão Preto, PAHO/WHO Collaborating Centre for Nursing Research Development, Ribeirão Preto, SP, Brazil; 2 Universidade Federal de São Carlos, São Carlos, SP, Brazil; 3 Scholarship holder at the Coordenação de Aperfeiçoamento de Pessoal de Nível Superior (CAPES), Brazil; 4 Hospital das Clínicas da Faculdade de Medicina de Ribeirão Preto, Ribeirão Preto, SP, Brazil; 5 Scholarship holder at the Conselho Nacional de Desenvolvimento Científico e Tecnológico (CNPq), Brazil

**Keywords:** Aged, Frailty, Geriatric, Multitasking Behavior, Cognitive Dysfunction, Cognition

## Abstract

**Objective:**

to analyze the influence of frailty and cognitive decline on dual-task performance in older adults.

**Methods:**

cross-sectional study carried out with older adults at a geriatrics outpatient clinic, in São Paulo. Sociodemographic data, cognitive performance, frailty phenotype, gait speed and dual-task assessments were used. The analysis was descriptive, combined with a multiple linear regression model.

**Results:**

219 older adults participated, with a mean age of 72.55 years, aged between 60 and 79 years (82.65%) and predominantly female (70.32%). Among them, 86 (39.27%) were frail and 123 (57.48%) presented cognitive decline. The mean time for simple walking was 15.95 (7.02) seconds; for the motor dual-task, 17.64 (8.44) seconds; and for the cognitive dual-task, 23.88 (11.87) seconds. Women without a partner, living with family, and with low education (0-4 years) required more time to perform both the cognitive and motor dual-tasks compared to the time for simple walking. Frail older adults exhibited impared gait performance, both in simple and dual motor/cognitive tasks.

**Conclusion:**

frail older adults experienced a significant decline in performance in simple gait and in dual motor/cognitive tasks, with no difference in performance between those with and without cognitive decline. Multidisciplinary interventions should focus on promoting the health of older adults.

## Highlights

**(1)** Frailty and sarcopenia are important syndromes that need to be assessed in older adults. **(2)** Older adults with T2DM have a higher vulnerability to developing frailty. **(3)** Nurses should implement preventive measures to address frailty and sarcopenia.

## Introduction

The proportion of older adults is increasing worldwide compared to other age groups^(1)^. The aging process and the manifestation of morbidities, when associated with disability, can influence the frailty process within the older adult population^(2)^. Frailty is a clinical condition that leads to increased vulnerability and is associated with negative outcomes such as falls, disability, hospitalization, and mortality^(3)^.

One of the dimensions used to evaluate the frailty phenotype is gait speed^(2)^ which is directly affected by the aging process^(4)^. As people age, there is a 20% decline in gait speed per decade and this reduction interferes with independence, thereby, impairing the performance of Activities of Daily Living (ADL)^(4)^. Gait disorders are prevalent among older adults and can lead to falls, immobility, institutionalization, and an increase in mortality^(5)^. Gait impairment may precede cognitive impairment and difficulty in ADLs and can also represent an early stage of the disability process^(5)^. However, the relationship between gait and cognition requires further research^(6)^.

In older adults, slower gait speed is associated with the risk of dementia and cognitive decline^(6)^. Gait speed can also differentiate healthy older adults from those with cognitive decline^(7)^. Additionally, the literature suggests the existence of a correlation between low cognitive performance and frailty in older adults^(8)^. A meta-analysis identified that the association between physical-functional decline and neurocognitive decline has become increasingly recurrent; it also demonstrated that the worsening of cognitive performance occurs during the transition from pre-frail to a frail condition, characterizing a “cognitively and physically frail” older adult phenotype^(7)^. Cognition and frailty are potential predictors of early mortality in the older adult population^(8)^. Gait speed can also differentiate older adults according to their frailty status (i.e., frail, pre-frail, non-frail) particularly when gait speed is reduced during dual-task activities^(9)^. Although changes in gait caused by dual-tasking are observed in healthy adults, such changes become more pronounced with age and in those with cognitive decline^(10)^. The severity of this change is directly related to cognitive decline^(10)^, likely due to reduced attention, which compromises gait stability^(11)^.

The association between gait during both simple and dual-task walking can help understand the deterioration of gait with aging and the connection between motor and cognitive function^(11)^. Dual-task walking may also be useful for detecting subtle and diverse gait deteriorations in aging^(9)^. Understanding these relationships will contribute to the design of interventions aimed at maintaining or recovering adequate gait patterns in the older adults. Therefore, the aim of this study was to analyze the influence of frailty and cognitive decline on dual-task performance in older adults.

Accordingly, the objective was to analyze the influence of frailty and cognitive decline on dual-task performance in community-dwelling older adults.

## Methods

### Study design, period, and location

This was an analytical, observational, and cross-sectional study conducted in accordance with the Strengthening the Reporting of Observational Studies in Epidemiology (STROBE)^(12)^ guidelines. The assessment protocol was developed between August and September 2019, with team training occurring in October 2019. Data collection began in November 2019 and continued until February 2020 at the School Health Center of the Faculty of Medicine, University of São Paulo, in Ribeirão Preto, São Paulo.

### Population and sample

To calculate the sample size, an *R*-squared coefficient of determination of .10 was considered for a multiple linear regression model with the predictors. The significance level, or Type I error rate, was set at α = .01, and the Type II error rate at β = .10, resulting in a predetermined statistical power of 90%. Using the PASS (Power Analysis and Sample Size) application, version 13, and inputting the aforementioned values, a minimum sample size of *n* = 206 was obtained. However, accounting for a 20% sampling loss (due to refusals to participate), the final number of attempted interviews was *n* = 258. The main dependent variable was the time recorded for the motor-cognitive dual-task gait test.

Participants were contacted prior to their medical consultation, and those who agreed to participate were evaluated after the consultation. The interviews were conducted by undergraduate, graduate, and post-doctoral students from the Research Center in Geriatrics and Gerontology (NUPEGG) at the Nursing School of Ribeirão Preto-USP. It should be emphasized that the interviewers were previously trained by the group leader to standardize the assessment techniques.

### Inclusion and exclusion criteria

The study population consisted of community-dwelling older adults who were users of the outpatient clinic of a Public Health Unit. To participate in the study, the following inclusion criteria needed to be met: being 60 years of age or over; of either sex; residing in a household in the city of Ribeirão Preto; being able to communicate verbally; and not having a diagnosis of dementia.

### Instruments for assessment

- *Socio-demographic Questionnaire*: Instrument developed by NUPEGG in 2006, which includes information on gender (male and female); age (in years); marital status (single, married, separated, divorced, or widowed); income of the older adult (net income in Brazilian Reais); schooling (in years of formal studies); number of children; number of people living in the same household; and retirement status (yes and no).
- *Cognitive Performance:* The Mini-Mental State Examination (MMSE) was used to assess this variable. The MMSE has been translated and validated into its Brazilian Portuguese version, with cut-off scores that vary according to the participant’s educational level. It has also been validated for hospital settings, clinical environments, and population studies^(13)^. Scores range from zero to 30 points; with the following cutoff points suggested by the authors: 20 for illiterate individuals, 24 for those with 1 to 4 years of schooling, 26.5 points for those with 5 to 8 years of schooling, 28 points for those with 9 to 11 years, and 29 points for those with more than 11 years of schooling^(13)^.
- *Frailty Phenotype*: For this assessment, participants were taken to an adapted room equipped with chairs, a scale, a stadiometer, and a manual hydraulic JAMAR dynamometer. According to the frailty phenotype^(2)^, the following measurements were assessed: body weight, (measured using a scale), fatigue, handgrip strength, gait speed, and level of physical activity. Fatigue was assessed using two questions from a depression screening scale, considered positive if participants reported needing a lot of effort to cope with tasks or being unable to carry out usual tasks for three days or more in the week. Handgrip strength was measured using a portable hydraulic dynamometer in the dominant hand, with results adjusted for gender and body mass index (BMI). Gait speed was measured by calculating the average time participants took to cover the distance of 4.6 m, with adjustments for sex and height. The average time was based on the speed of three trials. Finally, the level of physical activity was assessed using the International Physical Activity Questionnaire (IPAQ), proposed by the World Health Organization (WHO) in 1998. This instrument has been previously validated in several countries, including Brazil^(14)^, and is widely used worldwide^(15)^.
- *Gait Speed*: The Timed Up and Go test (TUG) was performed, where participants were instructed to rise from a 45 cm high chair with armrests, walk three meters at their usual speed, turn around, return to the chair, and sit down. To perform well, older adults need mobility, agility, and intact executive functions , making the TUG more than just a simple walking test^(15)^. The test starts with a “go” command and ends when the participant sits down again. The time taken to complete the three-meter course was recorded. The TUG has been validated in Brazil and is widely used worldwide^(14)^.
- *Dual-task*: The TUG can be quickly applied, is easy to be reproduced, and has also been associated with motor and/or cognitive secondary tasks (i.e. Dual-task TUG)^(16- 17)^. The Dual-Task TUG (DT-TUG) is a reliable biomarker for motor decline in the older adult population, as it identifies changes in gait parameters and aids in the differential diagnosis of Alzheimer’s Disease^(17)^. In this study, the cognitive dual-task (Cog-DT) was performed while the older adult walked the three-meter course and counted down numbers in increments of 3 (starting from a number between 80 and 99). For the motor dual-task (Mot-DT), participants walked a distance of 3 meters while holding a glass of water. The group means were compared, and a validation instrument was not used, as the means of the participants were compared according to the classification groups concerning frailty.

### Data analysis

The dependent variable in this study is the time required to complete the dual-task (Mot-DT). The independent variables include sociodemographic/clinical factors, cognitive function, frailty, and gait speed.

All variables were submitted to statistical analysis. For qualitative variables, absolute frequency measures were used; for categorical variables, relative measures; and for quantitative variables, measures of central tendency (mean, median, minimum, and maximum) and variability (amplitude and standard deviation) were employed. To relate the outcome variable with the exploratory variables, linear regression and Pearson’s correlation test was used. When the prerequisites for using parametric tests were not met, a non-parametric test (Mann-Whitney test) was applied. All tests adopted a significance level of *p* ≤ .05.To analyze the influence of sociodemographic and clinical characteristics, frailty, and cognitive decline on Mot-DT performance, a bivariate analysis was performed, including Student’s *t*-test for dichotomous predictors and Pearson’s correlations for quantitative predictors. The analysis of the simultaneous influence of sociodemographic and clinical characteristics, frailty, and cognitive decline predictors on dual-task (Mot-DT) performance and gait speed included multiple linear regression analysis. To analyze the influence of sociodemographic and clinical characteristics, frailty, and cognitive decline variables on gait speed, multiple linear regression was employed. For the analysis of the influence of sociodemographic and clinical characteristics, frailty, and cognitive decline on Cog-DT performance, bivariate analyses were used, including Student’s *t*-test for dichotomous predictors and Pearson’s correlations for quantitative predictors. The assessment of the simultaneous influence of sociodemographic and clinical characteristics, frailty, and cognitive decline predictors on dual-task (cognitive) performance and gait speed included multiple linear regression analysis.

To carry out simple and multiple linear regression, a literature search was conducted based on the data found, with this, the variables of interest were found to be: age, education, gender, arthritis, arterial hypertension, and frailty.

The SAS System for Windows (Statistical Analysis System), version 9.2 (SAS Institute Inc., 2002-2008, Cary, NC, USA), was used to conduct the statistical analyses.

### Ethical aspects

The Health Department of Ribeirão Preto authorized the investigation, and the research received approval (No 5.427.143), from the Research Ethics Committee (CEP) of the Nursing School of Ribeirão Preto, University of São Paulo, in accordance with Resolution 466/2012 of the National Health Council. Participants were informed about the research, and the assessment began only after they had signed the consent form. This manuscript is part of a larger project by one of the authors, entitled “Biomarkers related to fragility and sarcopenia in older adults”.

## Results

Participants (*n* = 219) were mostly white (69.41%) and female (70.32%), with a mean age of 72.55 (*SD* = 7.3) years; the predominant age range was between 60 and 79 years (82.55%). Of the total sample, 52.05% had a partner and the mean number of children was 3.04. It is important to highlight that 97.72% were users of the Brazilian National Health System. Schooling ranged from zero to more than 12 years, with a mean of 5.15 years of study (*SD* = 3.82).

Regarding income, the monthly mean was R$ 1,459.60 (*SD* = 934.55), with most participants (*n* = 155, 70.78%) receiving an amount above one minimum wage. Concerning the source of income, 174 participants (79.82%) did not have a retirement salary, while 179 received a pension (82.11%). A total of 202 older adults were still working (94.04%) and 215 reported receiving donations (98.62%). Additionally, 94.95% of the participants lived in a rented house.

Clinical characteristics were also assessed. Most of the participants were pre-frail (*n* = 131, 59.82%), presented cognitive decline (*n* = 123, 57.48%), reported having a good memory (*n* = 114, 52.05), and rated their memory to be as good as one year ago (*n* = 144, 65.75%). The mean gait speed of the older adults was 15.95 seconds (*SD* = 7.02), and the mean gait speeds for the Mot-DT and Cog-DT were 17.64 (*SD* = 8.44) and 23.88 (*SD* = 11.87) seconds, respectively. Table 1 presents the participants’ sociodemographic and clinical characteristics.

**Table 1 -:** Sociodemographic and clinical characteristics of the older adults living in the community (*n* = 219). Ribeirão Preto, SP, Brazil, 2022

Variable	Category	***n*** *	**%** ^†^
Gender	Female	154	70.32
	Male	65	29.68
Age	Mean (*SD*)^‡^	72.55 (7.3)	-
	Younger older adult 60-79	181	82.65
	Older older adult >80	38	17.35
Ethnicity	White	155	69.41
	Mixed	38	17.35
	Black	28	12.79
	Unknown	1	0.46
Living with a partner	No	105	47.95
	Yes	114	52.05
Health Services in use	SUS	214	97.72
	Private	4	1.83
	Pharmacy	1	0.46
Marital Status	Single	24	10.96
	Married	114	52.05
	Divorced	12	5.48
	Separated	8	3.65
	Widow	60	27.40
	Other	1	-
Years of Schooling	Mean (*SD*)^‡^	5.15 (3.82)	-
	0 – 4	131	61.21
	5 – 8	52	24.30
	9 – 11	14	6.54
	12 or more	17	7.94
Retirement Salary	Yes	44	20.18
	No	174	79.82
Pension	Yes	179	82.11
	No	39	17.89
Working Status	Active	202	94.04
	Not active	13	5.96
Monthly Income^ǁ^	R$1.039,00^ǁ^	58	26.48
	More than one minimum wage^ǁ^	155	70.78
Receive any donation	Yes	215	98.62
	No	3	1.38
Live in a rented house	Yes	207	94.95
	No	11	5.05
Number of children	Mean (*SD*)^‡^	3.04 (2.12)	-
Living Arrangement	Alone	32	14.61
	With a partner	66	30.13
	Other	119	54.33
Frailty	Non-Frail	2	0.91
	131	59.82
	86	39.27
	Pre-Frail		
	Frail		
Cognitive Decline	Without Cognitive Decline	91	42.52
	With Cognitive Decline	123	57.48
Memory self-assessment	Excellent	10	4.57
	Very Good	24	10.96
	Good	114	52.05
	Regular	58	26.48
	Bad	12	5.48
	Terrible	1	0.46
Memory, One Year Comparison	Better	21	9.59
	Same	144	65.75
	Worse	54	24.66
Variable		**Mean(*SD*)** ^‡^	
Gait Speed (seconds)		15.95 (7.02)	
Mot-DT (seconds)^§^		17.64 (8.44)	
Cog-DT (seconds)^¶^		23.88 (11.87)	

*n = Number of participants; ^†^% = Frequency; ^‡^ *SD* = Standard Deviation; ^§^Mot-DT= Motor Dual-Task; ^||^Minimum wage of the research country ^¶^Cog-DT= Cognitive Dual-Task

The results demonstrate that the presence of cognitive decline was not related to gait speed (*p* = .095), motor dual-task (*p* = .124), or cognitive dual-task (*p* = .069). Frailty, however, was an important variable regarding gait performance, both in single (*p* < .001) and dual-task (*p* < .001) gait. Consequently, according to the present analyses, it can be stated that frailty has a greater impact on dual-task performance and single gait speed compared to cognitive decline (Table 2).

Tables 3 and 4 present the results of the simple and multiple linear regression analyses (with Stepwise criterion for variable selection), respectively, to explore the relationship between the variables of interest and gait speed.

**Table 2 -:** Comparison of gait speed, Mot-DT*, and Cog-DT^†^ between the categorical variables cognitive decline and frailty (*n* = 219). Ribeirão Preto, SP, Brazil, 2022

Comparison with Cognitive Decline (MMSE)^‡^									
Variable			***n*** ^§^	Mean	***SD*** ^||^	Median	**Z** ^¶^	***p*** ^**^	
Without Decline		Gait Speed	91	15.02	5.69	13.50	1.67	*p* = .095	
		Mot-DT^*^	91	16.57	6.41	15.00	1.54	*p* = .124	
		Cog-DT^†^	91	22.42	9.40	20.00	1.82	*p* = .069	
With Decline		Gait Speed	123	16.69	7.93	14.50			
		Mot-DT^*^	123	18.51	9.75	16.70			
		Cog-DT^†^	123	25.15	13.47	22.00			
**Comparison with Frailty**									
Variable			***n*** ^§^	Mean	***SD*** ^||^	Median	*Z* ^¶^	***p*** ^**^	Comparison
Non-Frail	Gait Speed		2	10.00	0.71	10.00	16.34	*p* < .001	1≠3
(1)	Mot-DT*		2	12.00	1.41	12.00	26.81	*p* < .001	1≠3
	Cog-DT^†^		2	18.50	6.36	18.50	15.43	*p* < .001	1≠3
Pre-Frail	Gait Speed		131	14.84	5.86	13.50			
(2)	Mot-DT*		131	15.83	6.25	15.00			
	Cog-DT^†^		131	21.62	8.30	20.00			
Frail	Gait Speed		86	17.78	8.23	15.75			
(3)	Mot-DT*		86	20.53	10.43	18.00			
	Cog-DT^†^		86	27.45	15.29	23.50			
**Comparison with Frailty (Grouped)**									
	Variable	***n*** ^§^	Mean	***SD*** ^||^	Median	***Z*** ^¶^	***p*** ^**^		
Non-Frail/Pre-Frail	Gait Speed		133	14.76	5.85	13.00	3.69	*p* < .001	
	Mot-DT*		133	15.77	6.22	15.00	5.07	*p* < .001	
	Cog-DT^†^		133	21.57	8.26	20.00	3.90	*p* < .001	
Frail	Gait Speed		86	17.78	8.23	15.75			
	Mot-DT*		86	20.53	10.43	18.00			
	Cog-DT^†^		86	27.45	15.29	23.50			

*Mot-DT = Motor Dual Task; ^†^Cog-DT = Cognitive Dual Task; ^‡^MMSE = Mini Mental State Examination; ^§^ *n* = Number of Participants; ^||^ *SD* = Standard Deviation; ^¶^ *Z* = *Z* statistic of the test; **p-value referring to the Mann-Whitney test for comparison of variables between 2 groups; ^††^ *p*-value referring to the Kruskal-Wallis’ test for comparison of variables between 3 or more groups.

**Table 3 -:** Simple linear regression analysis for gait speed (*n* = 219). Ribeirão Preto, SP, Brazil, 2022

Gait Speed				
Variable	Categories	Beta* (se)^†^	***p*** ^‡^	***R*** ^2§^
Gender	Male (ref.)^ǁ^	-	-	-
	Female	30.87 (9.15)	< .001	.0498
Age group	60-79 years (ref.)^ǁ^	-	-	-
	≥ 80 years	47.95 (10.85)	< .001	.0826
Age (in years)	-	0.29 (0.06)	< .001	.0851
Schooling	0-4 years (ref.)^ǁ^	-	-	-
	5-8 years	-24.15 (10.05)	.017	-
	9-11 years	-51.85 (17.24)	.003	-
	≥ 12 years	-53.11 (15.80)	< .001	.0889
Frailty	Non-Frail/Pre-Frail (ref.)^ǁ^	-	-	-
	Frail	32.30 (8.50)	< .001	.0624
Cognitive Decline	No (ref.)^ǁ^	-	-	-
	Yes	14.80 (8.78)	.093	.0132
Arterial Hypertension	No (ref.)^ǁ^	-	-	-
	Yes	34.11 (8.55)	< .001	.0683
Arthritis	No (ref.)^ǁ^	-	-	-
	Yes	24.75 (8.44)	.004	.0382
**Motor Dual-Task**								
Variable	Categories	Beta* (se)^†^	p^‡^	R^2§^
Gender	Male (ref.)^ǁ^	-	-	-
	Female	20.14 (9.27)	.031	.0213
Age group	60-79 years (ref.)^ǁ^	-	-	-
	≥ 80 years	61.55 (10.51)	< .001	.1365
Age (in years)	-	0.37 (0.06)	< .001	.1341
Schooling	0-4 years (ref.)^ǁ^	-	-	-
	5-8 years	-28.83 (9.75)	.004	-
	9-11 years	-67.05 (16.73)	< .001	-
	≥ 12 years	-65.94 (15.34)	< .001	.1399
Frailty	Non-Frail/Pre-Frail (ref.)^ǁ^	-	-	-
	Frail	44.34 (8.24)	< .001	.1178
Cognitive Decline	No (ref.)^ǁ^	-	-	-
	Yes	13.64 (8.78)	.122	.0113
Arterial Hypertension	No (ref.)^ǁ^	-	-	-
	Yes	35.23 (8.52)	<.001	.0730
Arthritis	No (ref.)^ǁ^	-	-	-
	Yes	14.78 (8.53)	.084	.0136
**Cognitive Dual-Task**								
**Variable**	**Categories**	Beta* **(se)** ^†^	***p*** ^‡^	***R*** ^2§^
Gender	Male (ref.)^ǁ^	-	-	-
	Female	28.84 (9.17)	.002	.0435
Age group	60-79 years (ref.)^ǁ^	-	-	-
	≥ 80 years	43.38 (10.93)	< .001	.0677
Age (in years)	-	0.30 (0.06)	< .001	.0872
Schooling	0-4 years (ref.)^ǁ^	-	-	-
	5-8 years	-37.49 (9.58)	< .001	-
	9-11 years	-75.02 (16.44)	< .001	-
	≥ 12 years	-60.43 (15.07)	< .001	.1614
Frailty	Non-Frail/Pre-Frail (ref.)^ǁ^	-	-	-
	Frail	34.17 (8.46)	< .001	.0699
Cognitive Decline	No (ref.)^ǁ^	-	-	-
	Yes	15.89 (8.72)	.070	.0154
Arterial Hypertension	No (ref.)^ǁ^	-	-	-
	Yes	28.06 (8.65)	.001	.0463
Arthritis	No (ref.)^ǁ^	-	-	-
	Yes	14.19 (8.54)	.098	.0125

*Beta = Value of the estimate or angular coefficient (slope) on the regression line; ^†^se: Standard error of beta; ^‡^ *p*= Value; ^§^ *R* ^2^= Coefficient of determination (% variability of the response variable explained by the independent variable); ^ǁ^Ref = Reference; Variables without normal distribution were transformed into ranks

**Table 4 -:** Multiple linear regression analysis for gait speed (n = 219). Ribeirão Preto, SP, Brazil, 2022

Gait Speed				
Selected Variables	Categories	Beta* (se)^†^	***p*** ^‡^	***R*** ^2§^
Age Group	60-79 years (ref.)^§^	-	-	-
	≥ 80 years	41.56 (10.22)	<.001	.0845
Arterial Hypertension	No (ref.)^ǁ^	-	-	-
	Yes	26.27 (7.99)	.001	.0709
Gender	Male (ref.)^ǁ^	-	-	-
	Female	25.02 (8.71)	.005	.0473
Frailty	Non-Frail/Pre-Frail (ref.)^ǁ^	-	-	-
	Frail	20.11 (8.01)	.013	.0356
Schooling	0-4 years (ref.)^ǁ^	-	-	-
	5-8 years	-13.66 (9.19)	.139	-
	9-11 years	-26.02 (15.84)	.102	-
	≥ 12 years	-41.92 (14.30)	.004	.0367
Arthritis	No (ref.)^ǁ^	-	-	-
	Yes	17.14 (7.97)	.033	.0160
**Motor Dual-Task**				
**Selected Variables**	**Categories**	Beta* **(se)** ^†^	***p*** ^‡^	***R*** ^2§^
Age Group	60-79 years (ref.)^ǁ^	-	-	-
	≥ 80 years	48.62 (9.58)	< .001	.1376
Frailty	Non-Frail/Pre-Frail (ref.)^ǁ^	-	-	-
	Frail	30.84 (7.49)	< .001	.0851
Arterial Hypertension	No (ref.)^ǁ^	-	-	-
	Yes	28.52 (7.48)	< .001	.0561
Schooling	0-4 years (ref.)^ǁ^	-	-	-
	5-8 years	-14.49 (8.64)	.095	-
	9-11 years	-38.22 (14.90)	.011	-
	≥ 12 years	-52.75 (13.45)	< .001	.0689
Gender	Male (ref.)^ǁ^	-	-	-
	Female	20.16 (7.86)	.011	.0202
**Cognitive Dual-Task**				
**Selected Variables**	**Categories**	Beta*(se) ^†^	***p*** ^‡^	***R*** ^2§^
Frailty	Non-Frail/Pre-Frail (ref.)^ǁ^	-	-	-
	Frail	23.32 (7.92)	.004	.0734
Schooling	0-4 years (ref.)^ǁ^	-	-	-
	5-8 years	-28.24 (9.13)	.002	-
	9-11 years	-54.21 (15.75)	< .001	-
	≥ 12 years	-50.63 (14.22)	< .001	.1322
Gender	Male (ref.)^ǁ^	-	-	-
	Female	25.44 (8.31)	.003	.0337
Age Group	60-79 years (ref.)^ǁ^	-	-	-
	≥ 80 years	28.45 (10.13)	.006	.0255
Arterial Hypertension	No (ref.)^ǁ^	-	-	-
	Yes	19.81 (7.90)	.013	.0218

*Beta = Value of the estimate or angular coefficient (slope) on the regression line; ^†^se = Standard error of beta; ^‡^ *p*= Value; ^§^ *R* ^2^= Coefficient of determination; ^ǁ^Ref = Reference; Stepwise criterion for selecting variables; Total *R* ^2^: .2909; Intercept (se): 60.70 (10.00); *p*< .001; Variables without normal distribution were transformed into ranks

The simple linear regression analysis showed that being female, being 80 years of age or over (*p* < .001), having 0 to 4 years of education (*p* < .001), being frail (*p* < .001), having arterial hypertension (*p* < .001), and having arthritis (*p* < .004), were factors that worsened gait speed performance in the older adults. Those with the highest value for gait speed were: aged ≥ 80 years, female, with schooling of 0-4 years, with arterial hypertension, with arthritis, and with frailty.

Regarding the Mot-DT, the simple linear regression analysis showed that being 80 years of age or over (*p* < .001), having 0 to 4 years of education (*p* < .001), frailty (*p* < .001) and arterial hypertension (*p* < .001) were associated with worse performance in the motor dual-task.

Similarly, the simple linear regression analysis demonstrated that older adults aged 80 years or over (*p* < .001), with 0 to 4 years of education (*p* < .001), frailty (*p* < .001), and arterial hypertension (*p* = .001), also performed worse in the cognitive dual-task. Age (*p* < .001), gender (*p* = .005), education (*p* = .004), arterial hypertension (*p* = .001), arthritis (*p* = .033), and frailty (*p* = .013), presented a significant relationship with gait speed.

Based on the results of the multiple analysis, age (*p* < .001), education (*p* < .001), gender (*p* = .011), frailty (*p* < .001), and arterial hypertension (*p* < .001) presented a significant relationship with motor dual-task performance. The older adults who took the longest time to perform Mot-DT were: those aged 80 years or over, with 0-4 years of education, female, frail, and with arterial hypertension.

In the multiple analysis, frailty (*p* = .004), education (*p* < .001), gender (*p* = .003), age (*p* = .006), and arterial hypertension (*p* = .013) presented a significant relationship with Cog-DT performance. The older adults who took the longest time to perform the Cog-DT were those with frailty, 0-4 years of education, female, aged 80 years or over, and with arterial hypertension.

## Discussion

This study aimed to analyze the influence of frailty and cognitive decline on the performance of dual tasks in older adults, considering that autonomy and independence are essential aspects of people’s lives.

The mean age of the older adults was 72.55 years, with the most prevalent age group being younger older adults, aged between 60 and 79 years. This age group is one of the most prominent in Brazil^(4)^, with individuals aged 60 to 79 years representing 87.5% of the older adult population; the same trend is observed in low and middle-income countries.. Additionally, greater participation of women was observed in this study, which aligns with the existing literature. The demographic transition phenomenon, where the number of women aged 60 or over surpasses that of men, is referred to as the feminization of aging^(18)^. In this study, the mean schooling of the sample was 5.15 years. Other studies reported similar findings, with mean schooling of 5.42 years and 5.00 years, respectively. It is important to highlight that low education is directly related to frailty in older adults^(3)^ .The prevalence of frailty in the Brazilian population has not yet been adequately estimated, and the cutoff points for the items on frailty scales need to be adapted to parameters suited to this population^(18)^. This study identified that 39.27% of the older adults were frail. Similarly, a previous study^(1)^ that evaluated 360 older adults aged 65 years and over found a frailty prevalence of 48.7%. Additionally, 48.7% of the sample was non-frail and 32.2% were classified as pre-frail^(1)^. It is important to emphasize that 59.2% of the older adults in this study were classified as pre-frail, which is a reversible condition. This highlights the importance of implementing strategies to prevent frailty in both men and women, with a particular emphasis on preventing chronic conditions, which is especially critical for women^(19)^. Another important aspect is the low percentage of non-frail older adults. One possible explanation for this finding is that the study participants were individuals who had sought healthcare services, where data collection took place, and may have already had some health complaint or diagnosed conditions, such as hypertension and arthritis, both of which were present in this sample. Consequently, these individuals may have already experienced health limitations that were reflected in the five factors indicative of frailty. A Brazilian study involving 140 older adults with and without diabetes mellitus, found that frailty was more prevalent among older adults with type 2 diabetes mellitus compared to those without the disease (*p* = .00)^(20)^.

Furthermore, it is important to highlight that the prevalence of frailty in the older adult population varies significantly due to the use of different scales, the varying profiles of the older adults assessed, and the location of the study, among other factors.

Gait speed is a key component of the frailty construct^(2)^. Gait performance differs significantly for frail older adults, both with and without cognitive decline when compared to a control group. However, no significant differences have been found between these frail groups during both habitual walking and dual-task tests^(18)^. The absence of differences in gait parameters between frail individuals with and without cognitive decline, in both single and dual tasks, demonstrates the close relationship between these two conditions and further reinforces the idea that cognitive impairment and frailty share a common etiology^(18)^.In the present study, cognitive decline had a prevalence of 57.48% occurring in a heterogeneous and individual manner. Factors such as age, education, and functional capacity may be directly related to the prevalence of cognitive impairment in older adults^(8, 21)^.

Although no relationship was identified between cognitive decline and decreased gait speed, older adults with higher cognitive scores demonstrated better performance in gait speed in the dual tasks assessed. It is well-established that cognition and gait are closely related^(5)^. Recent evidence further indicates that cognitive impairment and falls are also linked It shows that walking impairments and falls are more common among individuals with dementia and that the prevalence of such impairments and falls increases with the severity of the cognitive decline^(21)^.Gait speed in dual tasks cannot differentiate individuals with preserved cognition from those with mild cognitive impairment, though it can specifically distinguish patients with Alzheimer’s disease from other cognitive profiles^(21)^.

The close relationship between cognition and gait arises from the shared brain structures involved in performing two tasks simultaneously. Dual-task methodologies create an environment that facilitates the manipulation of motor and cognitive tasks, which allows for the assessment of the cognitive-gait relationship^(22)^. Adding a processing task, such as problem-solving (e.g., counting down in increments to of 3), attention tasks, or visual demand during walking (e.g., carrying a glass) may impair gait performance, cognitive functioning, or both^(23)^.The findings of this study indicate that cognitive decline was not associated with worse performance in gait speed or in dual tasks, whether motor and cognitive. However, it was observed that the higher the cognitive score of the older adults, the better their performance in the assessed gait time.

Analyzing gait as a complex task can be advantageous for the health of older adults, as it can enhance its validity as an early marker for identifying cognitive decline^(24)^. Gait speed can serve as an indicator of cognitive function and the individual’s overall health status^(6)^.

The development of instruments that assess deficits related to both physical and cognitive frailty is crucial, given the association between the frailty syndrome and cognition in older adults. However, little is known about how much these factors influence dual-task performance^(24)^. Some studies have examined the influence of frailty syndrome, physical activity, and cognition in TUG and TUG-DT tests, comparing the performances of frail older adults. The findings indicated no cognitive differences between groups, however, the non-frail group completed the TUG test faster than the frail group. Regarding the TUG-DT, cognition, and age influenced the time taken to complete the task, however, no significant differences were observed between the groups. This suggests that frail older adults performed worse in the TUG when compared to non-frail participants, while the dual-task test did not distinguish frail from non-frail individuals, regardless of their cognitive performance^(23)^.Changes in gait speed during dual-task performances are sensitive indicators and can distinguish healthy individuals from those with cognitive impairment. Gait speed tends to be slower in older people with lower levels of education^(6, 24)^. Being female, single, and having low income are additional characteristics associated with slower gait speed, and individuals in this grou also tend to have lower cognitive function scores. Studies have found a correlation between slower gait speed and decreased cognitive function, leading to recommendations that geriatric rehabilitation programs include not only gait training and leg muscle strengthening but also cognitive training particularly targeting frontal and general cognitive abilities to improve gait performance^(6, 23- 24)^.

Performing secondary tasks while walking reduces gait speed, which suggests that gait relies on cognitive function^(5, 9)^. However, the decline in gait speed may also be influenced by the frailty status of older adults^(6)^.The relationship between cognition and gait during dual-task walking can serve as a valuable tool for monitoring even subtle gait deterioration throughout aging. It is evident that a cognitive challenge during motor tasks affect performance, and the ability to manage dual tasks diminishes progressively with aging, although the specific reasons for this decline remain unclear^(9)^.In the present study, the presence of arterial hypertension was found to negatively impact participants’ gait performance. Furthermore, gait speed itself is regarded as an early predictor of cardiovascular disease^(6)^. A study conducted in Taiwan explored the association between walking speed and the risk of cardiovascular disease among middle-aged and elderly individuals in the community. The findings suggested that slow gait speed was linked to an increased risk of cardiovascular disease among middle-aged adults, though not among older adults^(6)^. In this sense, our results align with those of a recent study^(6)^, in which gait speed performance was notably worse in older adults with more advanced age, lower levels of education, lower cognitive scores, and the presence of arterial hypertension.

This study underscores the potential for expanding the understanding of gait in older adults, recognizing it as an important indicator in assessing their overall health. Healthcare providers should carefully consider the clinical history and anamnesis during evaluations, as these factors represent risk parameters for frailty. The concept of the frailty phenotype used by the authors primarily focuses on physical aspects, and while gait is physical autonomy, its outcomes are influenced by various health dimensions, including nutrition. The assessment of frailty is gaining traction in both research and clinical practice, highlighting the urgent need for standardized protocols to guide healthcare providers, especially in the field of gerontology, enabling nurses and other practitioners to make informed assessments. This study has some limitations. Differences exist in the standardization of gait and cognitive assessments described in the literature. For example, in gait assessments, studies utilizing the TUG test often do not adhere to the specific distance used in the present study, complicating the comparison of findings across populations. Similarly, cognitive assessment, is carried out using a range of instruments, making it challenging to compare performance across different measures. Despite these limitations, studies should continue using various assessment instruments where appropriate, as this ensures the reliability and reproducibility for assessments conducted in healthcare settings. The aim is to detect deficits early to identify needs, organize, implement, and continually re-evaluate the efficiency of interventions, with the goal of improving the quality of life of the older adults. This process is fundamental and integrates the skills and competencies of healthcare providers. In doing so, it will contribute to improving the implementation of targeted interventions that enhance gait performance and/or maintain cognition function in the older adults.

## Conclusion

Based on the proposed objective, through the analysis of the influence of frailty and cognitive decline on dual-task performance in older adults, this study demonstrated that frailty exerts a greater interference on dual-task performance compared to cognitive performance. It is important to highlight that the strong association between gait and cognition has been described in many studies. Additionally, it is worth noting that older adults with gait deficits face an increased risk of developing cognitive deficits, and these cognitive deficits are associated with worsening gait performance.
