# Supplementary material for: Influence of frailty and cognitive decline on dual task performance in older adults: an analytical cross-sectional study
Source: Rev Lat Am Enfermagem. 2025 Feb 17;33:e4485. doi: 10.1590/1518-8345.7159.4485 (PMC11835002; doi:10.1590/1518-8345.7159.4485)
Supplement: Translation

# Translation: Influência da fragilidade e do declínio cognitivo na dupla tarefa de idosos: estudo transversal analítico*

**DOI:** 10.1590/1518-8345.7159.4486

**Authors:** Francine Golghetto Casemiro, Lucas Pelegrini Nogueira de Carvalho, Fernanda de Brito Matiello, Marcela Cristina Resende, Rosalina Aparecida Partezani Rodrigues

**Affiliations:** 1 Universidade de São Paulo, Escola de Enfermagem de Ribeirão Preto, Centro Colaborador da OPAS/OMS para o Desenvolvimento da Pesquisa em Enfermagem, Ribeirão Preto, SP, Brasil; 2 Universidade Federal de São Carlos, São Carlos, SP, Brasil; 3 Bolsista da Coordenação de Aperfeiçoamento de Pessoal de Nível Superior (CAPES), Brasil; 4 Hospital das Clínicas da Faculdade de Medicina de Ribeirão Preto, Ribeirão Preto, SP, Brasil; 5 Bolsista do Conselho Nacional de Desenvolvimento Científico e Tecnológico (CNPq), Brasil

**Keywords:** Idoso, Fragilidade, Geriatria, Comportamento Multitarefa, Disfunção Cognitiva, Cognição

## Abstract

**Objetivo:**

analisar a influência da fragilidade e do declínio cognitivo no desempenho da dupla tarefa em idosos.

**Método:**

estudo transversal, realizado com idosos em um ambulatório de geriatria, em São Paulo. Foram utilizados dados sociodemográficos e outros sobre desempenho cognitivo, fenótipo de fragilidade, velocidade da marcha e dupla tarefa. A análise foi descritiva e o modelo de regressão linear múltipla.

**Resultados:**

participaram 219 idosos, com média de idade de 72,55 anos, faixa etária entre 60 e 79 anos (82,65%), predomínio de idosos frágeis do sexo feminino (70,32%, 86 (39,27%) e 123 (57,48%) apresentaram declínio cognitivo. O tempo médio de caminhada simples foi de 15,95 (7,02) segundos, na dupla tarefa motora foi 17,64 (8,44) e na dupla tarefa cognitiva foi 23,88 (11,87). Idosas sem companheiro, morando com a família e com baixa escolaridade (0-4 anos) necessitaram de mais tempo para realizar a dupla tarefa cognitiva e motora, em comparação ao tempo para caminhada simples. Idosos frágeis demonstraram piora no desempenho da marcha, tanto nas tarefas motoras/cognitivas simples quanto nas duplas.

**Conclusão:**

idosos frágeis apresentaram piora significativa no desempenho na marcha simples e nas tarefas motoras/cognitivas duplas e aqueles com e sem declínio cognitivo não diferiram. Intervenções multidisciplinares devem ter como objetivo promover a saúde do idoso.

## Destaques

**(1)** A fragilidade e a sarcopenia são síndromes importantes que precisam ser avaliadas em idosos. **(2)** Idosos com DM2 apresentam maior vulnerabilidade para desenvolver fragilidade. **(3)** Os enfermeiros devem implementar medidas preventivas para enfrentar a fragilidade e a sarcopenia.

## Introdução

A proporção de idosos cresce mundialmente quando comparada a outras faixas etárias^(1)^. O processo de envelhecimento e a manifestação de morbidades, quando associados à incapacidade, podem influenciar o processo de fragilidade na população idosa^(2)^. A fragilidade é uma condição clínica que leva ao aumento da vulnerabilidade e está associada a desfechos negativos como quedas, incapacidade, hospitalização e mortalidade^(3)^.

Uma das dimensões para avaliar o fenótipo de fragilidade é a velocidade da marcha^(2)^, a qual é diretamente afetada pelo processo de envelhecimento^(4)^. À medida que as pessoas envelhecem, há declínio de 20% na velocidade da marcha por década e essa redução interfere na independência, o que, por sua vez, prejudica o desempenho das Atividades da Vida Diária (AVDs)^(4)^. Os distúrbios da marcha são prevalentes em idosos e podem levar a quedas, imobilidade, institucionalização e aumento da mortalidade^(5)^. O comprometimento da marcha pode preceder o comprometimento cognitivo e a dificuldade nas AVDs, além de representar um estágio inicial do processo de incapacidade^(5)^. No entanto, a relação entre marcha e cognição ainda precisa ser melhor investigada^(6)^.

Em adultos mais velhos, a velocidade mais lenta da marcha está associada ao risco de demência e declínio cognitivo^(6)^. Além disso, pode diferenciar idosos saudáveis daqueles com declínio cognitivo^(7)^. A literatura sugere a existência de correlação entre baixo desempenho cognitivo e condição de fragilidade em idosos^(8)^. Por outro lado, meta-análise identificou recorrência cada vez maior da associação entre declínio físico-funcional e declínio neurocognitivo, bem como piora no desempenho cognitivo durante a transição da condição pré-frágil para a condição frágil, o que caracteriza um fenótipo de idoso “cognitiva e fisicamente frágil”^(7)^. Cognição e fragilidade são potenciais preditores de mortalidade precoce na população idosa^(8)^. Finalmente, a velocidade da marcha também pode diferenciar os idosos quanto ao estado de fragilidade (ou seja, frágil, pré-frágil, não frágil) quando há redução dessa velocidade durante a dupla tarefa^(9)^.

Embora alterações na marcha causadas pela dupla tarefa sejam observadas em adultos saudáveis, tal condição aumenta com a idade e em pessoas com declínio cognitivo^(10)^. A intensidade dessa alteração está diretamente relacionada ao declínio cognitivo^(10)^, e isso se deve à redução da atenção, que compromete a estabilidade da marcha^(11)^.

A associação da marcha tanto na caminhada simples quanto na caminhada de dupla tarefa pode ajudar a entender a deterioração da marcha no envelhecimento e a ligação entre a função motora e cognitiva^(11)^. A caminhada de dupla tarefa também pode ser útil para monitorar deteriorações sutis e diversas da marcha no envelhecimento^(9)^; entender tais relações contribuirá para o planejamento de intervenções capazes de manter ou recuperar padrões adequados de marcha em idosos. Portanto, o objetivo deste estudo é analisar a influência da fragilidade e do declínio cognitivo no desempenho de dupla tarefa em idosos.

Como objetivo, definiu-se analisar a influência da fragilidade e do declínio cognitivo no desempenho de dupla tarefa em idosos que vivem na comunidade.

## Método

### Desenho, período e local do estudo

Trata-se de um estudo analítico, observacional e transversal, conduzido de acordo com as diretrizes do *Strengthening the Reporting of Observational Studies in Epidemiology* (STROBE)^(12)^. O protocolo de avaliação foi desenvolvido entre agosto e setembro de 2019. O treinamento da equipe ocorreu em outubro de 2019, e a coleta de dados teve início em novembro de 2019 e prosseguiu até fevereiro de 2020 no Centro de Saúde Escola da Faculdade de Medicina da Universidade de São Paulo, em Ribeirão Preto, São Paulo.

### População e amostra

Para calcular o tamanho da amostra, um coeficiente de determinação R-quadrado de 0,10 foi considerado em um modelo de regressão linear múltipla com os preditores. O nível de significância ou taxa de erro Tipo I foi definido em α = 0,01, e a taxa de erro Tipo II em β = 0,1, resultando em um poder estatístico predeterminado de 90%. Utilizando o aplicativo PASS (*Power Analysis and Sample Size*), versão 13, e inserindo os valores supracitados, obteve-se um tamanho amostral mínimo de n = 206. Entretanto, considerando uma perda amostral de 20% (recusas de participação), o número final de tentativas de entrevistas foi de n = 258. A principal variável dependente foi o tempo registrado para a execução do teste de marcha de dupla tarefa motora e cognitiva.

Os participantes foram contatados antes da consulta médica e aqueles que aceitaram participar passaram por avaliação após a consulta médica. As entrevistas foram realizadas por alunos de graduação, pós-graduação e pós-doutorado do Núcleo de Pesquisa em Geriatria e Gerontologia (NUPEGG) da Escola de Enfermagem de Ribeirão Preto-USP. Vale ressaltar que os entrevistadores receberam treinamento prévio do líder do grupo, com o objetivo de padronizar as técnicas de avaliação.

### Critérios de inclusão e exclusão

A população do estudo foi composta por idosos comunitários, usuários do ambulatório de uma unidade de saúde pública. Para participar do estudo, deveriam atender aos seguintes critérios: ter idade igual ou superior a 60 anos; ser de ambos os sexos; residir em domicílio no município de Ribeirão Preto; ser capaz de se comunicar verbalmente; e não possuir diagnóstico de demência.

### Instrumentos de avaliação:

- *Questionário sociodemográfico:* instrumento elaborado pelo NUPEGG em 2006, com informações sobre sexo (masculino e feminino), idade (em anos), estado civil (solteiro, casado, separado, divorciado ou viúvo), renda do idoso (renda líquida em reais); escolaridade (em anos de estudo formal), número de filhos, número de pessoas que vivem no mesmo domicílio e situação de aposentadoria (sim e não).
- *Desempenho cognitivo:* para avaliar essa variável, utilizou-se o Mini-Exame do Estado Mental (MEEM), traduzido e validado para sua versão em português brasileiro, em que as notas de corte variam de acordo com o nível educacional do participante. O MEEM também foi validado para ambientes hospitalares, contextos clínicos e estudos populacionais^(13)^. Seus escores variam de zero a 30 pontos; os pontos de corte sugeridos pelos autores foram os seguintes: 20 para analfabetos, 24 para aqueles com 1 a 4 anos de escolaridade, 26,5 pontos para pessoas com 5 a 8 anos de escolaridade, 28 pontos para participantes entre 9 e 11 anos e 29 pontos para aqueles com mais de 11 anos de escolaridade^(13)^.
- *Fenótipo de fragilidade:* para esta avaliação, os participantes foram levados a uma sala adaptada com cadeiras, balança, estadiômetro e dinamômetro hidráulico manual JAMAR. De acordo com o fenótipo de fragilidade^(2)^, foram avaliadas as seguintes medidas: peso corporal, obtido por balança; fadiga; preensão manual; velocidade da marcha; e nível de atividade física. A fadiga foi avaliada por duas questões de uma escala de triagem de depressão. Considerou-se positiva se os participantes declarassem a necessidade de fazer muito esforço para lidar com as tarefas ou incapacidade de realizar suas tarefas habituais em três dias ou mais na semana. A força de preensão manual foi medida usando um dinamômetro hidráulico portátil na mão dominante. Os resultados foram ajustados para gênero e índice de massa corporal (IMC). Para a velocidade da marcha, calculou-se o tempo médio gasto pelos participantes para percorrer a distância de 4,6 m. Ajustes de acordo com sexo e altura também foram aplicados e calculou-se o tempo médio com base na velocidade de três tentativas. Por fim, avaliou-se o nível de atividade física pelo Questionário Internacional de Atividade Física (IPAQ), proposto pela Organização Mundial da Saúde (OMS) em 1998. Este instrumento foi previamente validado em vários países, inclusive no Brasil^(14)^, e tem sido amplamente utilizado em todo o mundo^(15)^.
- *Velocidade da marcha:* foi realizado o teste *Timed Up and Go* (TUG), e cada participante foi instruído a se levantar de uma cadeira de 45 cm de altura com braços, caminhar por uma distância de três metros em velocidade habitual, virar-se, retornar à cadeira e sentar-se. Para obter um bom desempenho, os idosos precisam de mobilidade, agilidade e ter suas funções executivas em boas condições, portanto o TUG não é um teste simples de caminhada^(15)^. O teste inicia com o comando “vai” e termina quando o idoso se senta novamente. Para essa avaliação, o tempo de execução do percurso durante os três metros foi registrado, validado no Brasil e tem sido amplamente utilizado no mundo^(14)^.
- *Dupla tarefa:* o TUG pode ser aplicado rapidamente, é fácil de ser reproduzido e também tem sido associado a tarefas secundárias motoras e/ou cognitivas (por exemplo, *Dual-task* TUG)^(16)^. O *Dual-Task* TUG (DT-TUG) é um biomarcador confiável para o declínio motor na população idosa, pois identifica alterações nos parâmetros da marcha e auxilia no diagnóstico diferencial da Doença de Alzheimer^(16)^. Neste estudo, a tarefa dupla cognitiva (DT-Cog) foi realizada enquanto o idoso caminhava o percurso de três metros e contava números de 3 em 3 (começando com um número entre 80 e 99). Para a tarefa dupla motora (DT-Mot), estabeleceu-se o seguinte objetivo: o idoso caminharia uma distância de 3 metros segurando um copo de água. Não foi utilizado um instrumento de validação, pois as médias dos participantes foram comparadas de acordo com os grupos de classificação quanto à fragilidade.

### Análise de dados

A variável dependente neste estudo é o tempo necessário para completar a tarefa dupla (Mot-DT). As variáveis independentes incluem fatores sociodemográficos, clínicos, função cognitiva, fragilidade e velocidade da marcha.

Todas as variáveis foram submetidas à análise estatística. Para variáveis qualitativas, foram utilizadas medidas de frequência absoluta; para variáveis categóricas, medidas relativas; e para variáveis quantitativas, medidas de tendência central (média, mediana, mínimo e máximo) e variabilidade (amplitude e desvio-padrão). Para relacionar a variável de desfecho com as variáveis exploratórias, optou-se por utilizar a regressão linear e o teste de correlação de Pearson. Quando os pré-requisitos para o uso de testes paramétricos não foram atendidos, aplicou-se um teste não paramétrico (Teste de Mann-Whitney). Todos os testes adotaram nível de significância de p≤0,05.

Para analisar a influência das características sociodemográficas e clínicas, fragilidade e declínio cognitivo no desempenho do DT-Mot, foi realizada uma análise bivariada incluindo o teste t de Student para preditores dicotômicos, bem como correlações de Pearson para preditores quantitativos. A análise da influência simultânea das características sociodemográficas e clínicas, fragilidade e preditores de declínio cognitivo na dupla tarefa (DT-Mot) e velocidade da marcha incluiu análise de regressão linear múltipla.

Para analisar a influência das características sociodemográficas e clínicas, fragilidade e variáveis de declínio cognitivo na velocidade da marcha, utilizou-se regressão linear múltipla. Para a análise da influência das características sociodemográficas e clínicas, fragilidade e declínio cognitivo no desempenho do DT-Cog, foram utilizadas análises bivariadas, incluindo o teste t de Student para preditores dicotômicos, bem como correlações de Pearson para preditores quantitativos. A avaliação da influência simultânea de características sociodemográficas e clínicas, fragilidade e preditores de declínio cognitivo na dupla tarefa (cognitiva) e velocidade da marcha incluiu análise de regressão linear múltipla.

Para realizar a regressão linear simples e múltipla, conduziu-se uma busca na literatura com base nos dados do presente estudo, de modo que as variáveis de interesse foram: idade, educação, gênero, artrite, hipertensão arterial e fragilidade. Utilizou-se o *SAS System for Windows* (*Statistical Analysis System*), versão 9.2 (*SAS Institute Inc.*, 2002-2008, Cary, NC, EUA), para executar análises estatísticas.

### Aspectos éticos

A Secretaria de Saúde de Ribeirão Preto autorizou a investigação e a pesquisa recebeu aprovação (5.427.143- aprovação) do Comitê de Ética e Pesquisa (CEP) da Escola de Enfermagem de Ribeirão Preto da Universidade de São Paulo, conforme Resolução 466/2012 do Conselho Nacional de Saúde. Os participantes foram informados sobre a pesquisa. A avaliação teve início somente após a assinatura do Termo de Consentimento Livre e Esclarecido. Este manuscrito faz parte de um grande projeto de um dos autores, intitulado “Biomarcadores relacionados à fragilidade e sarcopenia em idosos”.

## Resultados

Os participantes (n=219) eram majoritariamente brancos (69,41%) e do sexo feminino (70,32%), média de idade de 72,55 (DP=7,3) anos e predomínio da faixa etária entre 60 e 79 anos (82,55%). Do total da amostra, 52,05% tinham companheiro e a média de filhos chegou a 3,04. É importante destacar que 97,72% eram usuários do Sistema Único de Saúde. A escolaridade variou de zero a mais de 12 anos, com média de 5,15 anos de estudo (DP=3,82).

Em relação à renda, a média mensal foi de R$ 1.459,60 reais (DP=934,55), com a maioria dos participantes (n=155, 70,78%) recebendo valores acima de um salário-mínimo. Em relação à fonte de renda, 174 idosos (79,82%) não possuíam salário de aposentadoria e 179 recebiam pensão (82,11%). Um total de 202 idosos ainda estava trabalhando (94,04%) e 215 referiram receber doações (98,62%). Além disso, 94,95% dos participantes moravam em casa alugada.

Características clínicas também foram avaliadas. A maioria dos participantes era pré-frágil (n=131, 59,82%), com declínio cognitivo (n=123, 57,48%), referiu boa memória (n=114, 52,05) e classificou sua memória como boa há um ano (n=144, 65,75%). A velocidade média da marcha dos idosos foi de 15,95 segundos (DP=7,02) e as velocidades médias da marcha para Mot-DT e Cog-DT foram, respectivamente, 17,64 (DP=8,44) e 23,88 (DP=11,87). A Tabela 1 mostra as características sociodemográficas e clínicas dos participantes.

**Tabela 1 -:** Características sociodemográficas e clínicas dos idosos residentes na comunidade (n = 219). Ribeirão Preto, SP, Brasil, 2022

**Variável**	**Categoria**	n*	**%** ^†^
Gênero	Feminino	154	70,32
	Masculino	65	29,68
Idade	Média (=DP)^‡^	72,55 (7,3)	-
	Idoso mais jovem 60-79	181	82,65
	Idoso mais velho >80	38	17,35
Etnia	Branco	155	69,41
	Pardo	38	17,35
	Preto	28	12,79
	Desconhecido	1	0,46
Viver com um parceiro	Não	105	47,95
	Sim	114	52,05
Serviços de saúde utilizados	SUS	214	97,72
	Privado	4	1,83
	Farmácia	1	0,46
Estado civil	Solteiro	24	10,96
	Casado	114	52,05
	Divorciado	12	5,48
	Separado	8	3,65
	Viúvo	60	27,40
	Outro	1	-
Escolaridade	Média (=DP)^‡^	5,15 (3,82)	-
	0 – 4	131	61,21
	5 – 8	52	24,30
	09-11	14	6,54
	12 ou mais	17	7,94
Salário de aposentadoria	Sim	44	20,18
	Não	174	79,82
Pensão	Sim	179	82,11
	Não	39	17,89
Estado de trabalho	Ativo	202	94,04
	Inativo	13	5,96
Renda mensaǁ	R$1.039,00^ǁ^	58	26,48
	Mais do que um salário-mínimo	155	70,78
Recebe alguma doação	Sim	215	98,62
	Não	3	1,38
Vive em casa alugada	Sim	207	94,95
	Não	11	5,05
Número de filhos	Média (=DP)^‡^	3,04 (2,12)	-
Vivendo com	Sozinho	32	14,61
	Com um parceiro	66	30,13
	Outro	119	54,33
Fragilidade	Não frágil	2	0,91
	Pré-frágil	131	59,82
	Frágil	86	39,27
Declínio cognitivo	Sem declínio cognitivo	91	42,52
	Com declínio cognitivo	123	57,48
Autoavaliação de memória	Excelente	10	4,57
	Muito boa	24	10,96
	Bom	114	52,05
	Regular	58	26,48
	Ruim	12	5,48
	Terrível	1	0,46
Memória em comparação ao período de um ano	Bom	21	9,59
	Igual	144	65,75
	Pior	54	24,66
**Variável**		Média (DP)^‡^	
Velocidade de marcha (segundos)		15,95 (7,02)	
DT-Mot (segundos)^§^		17,64 (8,44)	
DT-Cog (segundos)^¶^		23,88 (11,87)	

*n = Número de participantes; ^†^% = Frequência; ^‡^DP = Desvio-padrão; ^§^DT-Mot = Dupla tarefa motora; ^||^Salário-mínimo do país da pesquisa; ^¶^DT-Cog = Dupla tarefa cognitiva

Os resultados demonstram que a presença de declínio cognitivo não está relacionada à velocidade da marcha (p=0,095), à dupla tarefa motora (p=0,124) ou à dupla tarefa cognitiva (p=0,069). A fragilidade, por outro lado, emergiu como uma variável importante em relação ao desempenho da marcha, tanto na marcha simples (p<0,001) quanto na dupla tarefa (p<0,001). Assim, de acordo com as presentes análises, pode-se afirmar que a fragilidade tem maior impacto no desempenho da dupla tarefa e na velocidade da marcha simples quando comparada ao declínio cognitivo (Tabela 2).

As Tabelas 3 e Tabelas 4 apresentam os resultados das análises de regressão linear simples e múltipla (com critério Stepwise para seleção de variáveis), respectivamente, para estudar a relação entre as variáveis de interesse e a velocidade da marcha.

**Tabela 2 -:** Comparação da velocidade da marcha, Mot-DT* e Cog-DT^†^ entre as variáveis categóricas declínio cognitivo e fragilidade (n = 219). Ribeirão Preto, SP, Brasil, 2022

**Comparação com declínio cognitivo (MMSE)** ^‡^									
	Variável		N^§^	Média	DP^||^	Mediana	Z^¶^	P**	
Sem declínio		Velocidade de marcha	91	15,02	5,69	13,50	1,67	p=0,095	
		DT-Mot*	91	16,57	6,41	15,00	1,54	p=0,124	
		DT-Cog^†^	91	22,42	9,40	20,00	1,82	p=0,069	
Com declínio		Velocidade de marcha	123	16,69	7,93	14,50			
		DT-Mot*	123	18,51	9,75	16,70			
		DT-Cog^†^	123	25,15	13,47	22,00			
**Comparação com fragilidade**									
	**Variável**		**N** ^§^	**Média**	**DP** ^||^	**Mediana**	**Z** ^¶^	p**	**Comparação**
Não frágil	Velocidade de marcha		2	10,00	0,71	10,00	16,34	p<0,001	1≠3
(1)	DT-Mot*		2	12,00	1,41	12,00	26,81	p<0,001	1≠3
	DT-Cog^†^		2	18,50	6,36	18,50	15,43	p<0,001	1≠3
Pré-frágil	Velocidade de marcha		131	14,84	5,86	13,50			
(2)	DT-Mot*		131	15,83	6,25	15,00			
	DT-Cog^†^		131	21,62	8,30	20,00			
Frágil	Velocidade de marcha		86	17,78	8,23	15,75			
**Comparação com fragilidade**									
(3)	DT-Mot*		86	20,53	10,43	18,00			
	DT-Cog^†^		86	27,45	15,29	23,50			
**Comparação com fragilidade (agrupada)**									
	**Variável**		**N** ^§^	**Média**	**DP** ^||^	**Mediana**	**Z** ^¶^	**p** ^††^	
Não frágil/Pré-frágil	Velocidade de marcha		133	14,76	5,85	13,00	3,69	p<0,001	
	DT-Mot*		133	15,77	6,22	15,00	5,07	p<0,001	
	DT-Cog^†^		133	21,57	8,26	20,00	3,90	p<0,001	
Frágil	Velocidade de Marcha		86	17,78	8,23	15,75			
	DT-Mot*		86	20,53	10,43	18,00			
	DT-Cog^†^		86	27,45	15,29	23,50			

*DT-Mot = Dupla Tarefa Motora; ^†^DT-Cog = Dupla Tarefa Cognitiva; ^‡^MMSE = Mini-Exame do Estado Mental; ^§^n = Número de participantes; ^||^DP= Desvio-padrão; ^¶^Z= Estatística Z do teste; **Valor de p referente ao teste de Mann-Whitney para comparação de variáveis entre 2 grupos; ^††^Valor de p referente ao teste de Kruskal-Wallis para comparação de variáveis entre 3 ou mais grupos

**Tabela 3 -:** Análise de regressão linear simples para velocidade da marcha (n = 219). Ribeirão Preto, SP, Brasil, 2022

Velocidade da marcha				
Variável	Categorias	Beta* (se)^†^	p^‡^	R2^§^
Gênero	Masculino (ref.)^ǁ^	-	-	-
	Feminino	30,87 (9,15)	<0,001	0,0498
Grupo etário	60-79 anos (ref.)^ǁ^	-	-	-
	≥80 anos	47,95 (10,85)	<0,001	0,0826
Idade (em anos)	-	0,29 (0,06)	<0,001	0,0851
Anos de educação	0-4 anos (ref.)^ǁ^	-	-	-
	5-8 anos	-24,15 (10,05)	0,017	-
	9-11 anos	-51,85 (17,24)	0,003	-
	≥12 anos	-53,11 (15,80)	<0,001	0,0889
Fragilidade	Não frágil/Pré-frágil (ref.)^ǁ^	-	-	-
	Frágil	32,30 (8,50)	<0,001	0,0624
Declínio cognitivo	Não (ref.)^ǁ^	-	-	-
	Sim	14,80 (8,78)	0,093	0,0132
Hipertensão arterial	Não (ref.)^ǁ^	-	-	-
	Sim	34,11 (8,55)	<0,001	0,0683
Artrite	Não (ref.)^ǁ^	-	-	-
	Sim	24,75 (8,44)	0,004	0,0382
**Dupla tarefa motora**				
Gênero	Masculino (ref.)^ǁ^	-	-	-
	Feminino	20,14 (9,27)	0,031	0,0213
Grupo etário	60-79 anos (ref.)^ǁ^	-	-	-
	≥80 anos	61,55 (10,51)	<0,001	0,1365
Idade (em anos)	-	0,37 (0,06)	<0,001	0,1341
Anos de educação	0-4 anos (ref.)^ǁ^	-	-	-
	5-8 anos	-28,83 (9,75)	0,004	-
	9-11 anos	-67,05 (16,73)	<0,001	-
	≥12 anos	-65,94 (15,34)	<0,001	0,1399
Fragilidade	Não frágil/Pré-frágil (ref.)^ǁ^	-	-	-
	Frágil	44,34 (8,24)	<0,001	0,1178
Declínio cognitivo	Não (ref.)^ǁ^	-	-	-
	Sim	13,64 (8,78)	0,122	0,0113
Hipertensão arterial	Não (ref.)^ǁ^	-	-	-
	Sim	35,23 (8,52)	<0,001	0,0730
Artrite	Não (ref.)^ǁ^	-	-	-
	Sim	14,78 (8,53)	0,084	0,0136
**Dupla tarefa cognitiva**				
Gênero	Masculino (ref.)^ǁ^	-	-	-
	Feminino	28,84 (9,17)	0,002	0,0435
Grupo etário	60-79 anos (ref.)^ǁ^	-	-	-
	≥80 anos	43,38 (10,93)	<0,001	0,0677
Idade (em anos)	-	0,30 (0,06)	<0,001	0,0872
Anos de educação	0-4 anos (ref.)^ǁ^	-	-	-
	5-8 anos	-37,49 (9,58)	<0,001	-
	9-11 anos	-75,02 (16,44)	<0,001	-
	≥12 anos	-60,43 (15,07)	<0,001	0,1614
Fragilidade	Não frágil/Pré-frágil (ref.)^ǁ^	-	-	-
	Frágil	34,17 (8,46)	<0,001	0,0699
Declínio cognitivo	Não (ref.)^ǁ^	-	-	-
	Sim	15,89 (8,72)	0,070	0,0154
Hipertensão arterial	Não (ref.)^ǁ^	-	-	-
	Sim	28,06 (8,65)	0,001	0,0463
Artrite	Não (ref.)^ǁ^	-	-	-
	Sim	14,19 (8,54)	0,098	0,0125

*Beta = Valor da estimativa ou coeficiente angular (inclinação) na reta de regressão; ^†^se = Erro-padrão do beta; ^‡^p = Valor; ^§^R2 = Coeficiente de determinação (% variabilidade da variável resposta explicada pela variável independente); ^ǁ^Ref= Referência. Variáveis sem distribuição normal foram transformadas em *ranks*

**Tabela 4 -:** Análise de regressão linear múltipla para velocidade da marcha (n = 219). Ribeirão Preto, SP, Brasil, 2022

Velocidade de marcha				
Variáveis selecionadas	Categorias	Beta* (se)^†^	p^‡^	R2^§^
Grupo etário	60-79 anos (ref.)^§^	-	-	-
	≥80 anos	41,56 (10,22)	<0,001	0,0845
Hipertensão arterial	Não (ref.)^ǁ^	-	-	-
	Sim	26,27 (7,99)	0,001	0,0709
Gênero	Masculino (ref.)^ǁ^	-	-	-
	Feminino	25,02 (8,71)	0,005	0,0473
Fragilidade	Não frágil/Pré-frágil (ref.)^ǁ^	-	-	-
	Frágil	20,11 (8,01)	0,013	0,0356
Anos de educação	0-4 anos (ref.)^ǁ^	-	-	-
	5-8 anos	-13,66 (9,19)	0,139	-
	9-11 anos	-26,02 (15,84)	0,102	-
	≥12 anos	-41,92 (14,30)	0,004	0,0367
Artrite	Não (ref.)^ǁ^	-	-	-
	Sim	17,14 (7,97)	0,033	0,0160
**Dupla tarefa motora**				
Grupo etário	60-79 anos (ref.)^ǁ^	-	-	-
	≥80 anos	48,62 (9,58)	<0,001	0,1376
Fragilidade	Não frágil/Pré-frágil (ref.)^ǁ^	-	-	-
	Frágil	30,84 (7,49)	<0,001	0,0851
Hipertensão arterial	Não (ref.)^ǁ^	-	-	-
	Sim	28,52 (7,48)	<0,001	0,0561
Anos de educação	0-4 anos (ref.)^ǁ^	-	-	-
	5-8 anos	-14,49 (8,64)	0,095	-
	9-11 anos	-38,22 (14,90)	0,011	-
	≥12 anos	-52,75 (13,45)	<0,001	0,0689
Gênero	Masculino (ref.)^ǁ^	-	-	-
	Feminino	20,16 (7,86)	0,011	0,0202
**Dupla tarefa cognitiva**				
Fragilidade	Não frágil/Pré-frágil (ref.)^ǁ^	-	-	-
	Frágil	23,32 (7,92)	0,004	0,0734
Anos de educação	0-4 anos (ref.)^ǁ^	-	-	-
	5-8 anos	-28,24 (9,13)	0,002	-
	9-11 anos	-54,21 (15,75)	<0,001	-
	≥12 anos	-50,63 (14,22)	<0,001	0,1322
Gênero	Masculino (ref.)^ǁ^	-	-	-
	Feminino	25,44 (8,31)	0,003	0,0337
Grupo etário	60-79 anos (ref.)^ǁ^	-	-	-
	≥80 anos	28,45 (10,13)	0,006	0,0255
Hipertensão arterial	Não (ref.)^ǁ^	-	-	-
	Sim	19,81 (7,90)	0,013	0,0218

*Beta = Valor da estimativa ou coeficiente angular (inclinação) na reta de regressão; ^†^se = Erro-padrão do beta; ^‡^p = Valor; ^§^R2 = Coeficiente de determinação; ^ǁ^Ref = Referência. Critério *stepwise* para seleção de variáveis. R2 total: 0,2909. Intercepto (se): 60,70 (10,00); p<0,001. Variáveis sem distribuição normal foram transformadas em *ranks*

A análise de regressão linear simples mostrou que ser do sexo feminino, ter 80 anos ou mais (p<0,001), possuir de 0 a 4 anos de estudo (p<0,001), ser frágil (<0,001), ter hipertensão arterial (<0,001) e artrite (p<0,004) são fatores que pioram o desempenho da velocidade da marcha em idosos. Os idosos com maior valor para velocidade da marcha foram aqueles com idade ≥80 anos, do sexo feminino, escolaridade de 0 a 4 anos, com hipertensão arterial e artrite e fragilidade.

Em relação ao DT-Mot, a análise de regressão linear simples evidenciou relação entre ter 80 anos ou mais (p<0,001), possuir entre 0 e 4 anos de estudo (p<0,001), ser frágil (p<0,001) e apresentar hipertensão arterial (p<0,001) com pior desempenho na tarefa motora dupla. Por outro lado, a análise de regressão linear simples mostrou que idosos com 80 anos ou mais (p<0,001), com 0 a 4 anos de estudo (p<0,001), frágeis (p<0,001) e com hipertensão arterial (p=0,001) apresentam pior desempenho na dupla tarefa cognitiva. Houve relação significativa entre idade (p<0,001), gênero (p=0,005), escolaridade (p=0,004), hipertensão arterial (p=0,001), artrite (p=0,033) e fragilidade (p=0,013) com a velocidade da marcha.

Com base nos resultados da análise múltipla, identifica-se relação significativa entre idade (p<0,001), escolaridade (p<0,001), gênero (p=0,011), fragilidade (p<0,001), hipertensão arterial (p<0,001) e dupla tarefa motora. Os idosos com maior tempo para realizar o Mot-DT foram aqueles com idade ≥80 anos, com 0-4 anos de estudo, do sexo feminino, frágeis e com hipertensão arterial.

Na análise múltipla, houve relação significativa entre fragilidade (p=0,004), escolaridade (p<0,001), sexo (p=0,003), idade (p=0,006) e hipertensão arterial (p=0,013) com o DT-Cog. Os idosos com maior tempo para realizar o DT-Cog foram aqueles com fragilidade, com 0-4 anos de estudo, do sexo feminino, com idade ≥80 anos e com hipertensão arterial.

## Discussão

O objetivo do presente estudo foi analisar a influência da fragilidade e do declínio cognitivo no desempenho de dupla tarefa em idosos, considerando que autonomia e independência são essenciais na vida das pessoas.

A média de idade dos participantes foi de 72,55 anos. Os idosos mais jovens, entre 60 e 79 anos, compuseram a faixa etária mais prevalente, a qual é também uma das mais proeminentes no Brasil^(4)^ e representa 87,5% dos idosos, tal como observado em países de baixa e média renda. Além disso, houve maior participação de mulheres neste estudo, o que novamente está de acordo com a literatura. O fenômeno resultante da transição demográfica, em que o número de mulheres com 60 anos ou mais se destaca do contingente masculino, é denominado de feminização da idade^(18)^. Neste estudo, a escolaridade média da amostra foi de 5,15 anos. Outros estudos encontraram escolaridade média de 5,42 anos e 5,0 anos, respectivamente, semelhante aos presentes achados. Faz-se importante mencionar que a baixa escolaridade está diretamente relacionada à fragilidade em idosos^(3)^.

A prevalência de fragilidade na população brasileira ainda não foi adequadamente estimada, e os pontos de corte dos itens que compõem as escalas devem ser adaptados aos parâmetros para essa população^(18)^. Este estudo identificou que 39,27% dos idosos eram frágeis. Da mesma forma, pesquisa^(1)^ avaliou 360 idosos com 65 anos ou mais e identificou prevalência de fragilidade em 48,7% da amostra estudada. Além disso, foram identificados 48,7% de idosos não frágeis e 32,2% de pré-frágeis^(1)^. Neste estudo, 59,2% dos idosos eram pré-frágeis, uma condição reversível. Isso ressalta a importância de estratégias voltadas à prevenção da fragilidade em ambos os sexos, enquanto a prevenção de condições crônicas é mais crucial em mulheres^(19)^. Outro ponto importante é o baixo percentual de idosos não frágeis, talvez porque os idosos que participaram da pesquisa procuraram os serviços de saúde onde ocorreu a coleta de dados já com alguma queixa de saúde ou diagnóstico de doença, como hipertensão e artrite, presentes nessa amostra. Consequentemente, esses indivíduos já teriam algumas limitações de saúde, que se refletem nos cinco fatores indicativos de fragilidade. Ademais, a prevalência de fragilidade na população idosa varia muito em virtude da diversidade de escalas utilizadas, dos diferentes perfis de idosos avaliados e dos locais dos estudos, por exemplo.

A velocidade da marcha é um componente do construto fragilidade^(2)^. O desempenho da marcha é significativamente diferente para idosos frágeis com e sem declínio cognitivo quando comparado ao grupo-controle; no entanto, não foram encontradas diferenças significativas entre esses grupos durante os testes de caminhada habitual e dupla tarefa^(18)^. A ausência de diferenças nos parâmetros da marcha entre os grupos frágeis com e sem declínio cognitivo, tanto em tarefas simples quanto em tarefas duplas, comprova a estreita relação entre ambos os transtornos e reforça a ideia de que comprometimento cognitivo e fragilidade compartilham da mesma etiologia^(18)^.

No presente estudo, o declínio cognitivo obteve prevalência de 57,48%. Ele ocorre de forma heterogênea e individual, mas fatores como idade, escolaridade e capacidade funcional podem estar diretamente relacionados à prevalência de comprometimento cognitivo em idosos^(8, 20)^.

Embora no presente estudo não tenha sido identificada relação entre declínio cognitivo e diminuição da velocidade da marcha, os idosos que obtiveram maiores escores cognitivos foram aqueles com melhor desempenho na velocidade da marcha em relação às tarefas duplas avaliadas. Sabe-se que cognição e marcha estão intimamente relacionadas^(5)^. Evidências recentes demonstram que comprometimento cognitivo e quedas também estão relacionados, bem como predomínio de comprometimentos na marcha e quedas em pessoas com demência e aumento da prevalência de comprometimentos na marcha e quedas com a gravidade do comprometimento cognitivo^(20)^.

A velocidade da marcha na tarefa dupla não consegue distinguir pessoas com cognição preservada daquelas com comprometimento cognitivo leve, embora permita diferenciar pacientes com doença de Alzheimer especificamente de outros perfis cognitivos^(20)^. A estreita relação entre cognição e marcha se deve ao compartilhamento de estruturas cerebrais durante a execução simultânea de duas tarefas. As metodologias de tarefa dupla fornecem um ambiente que permite a manipulação de tarefas motoras e cognitivas para avaliar a relação cognitivo-marcha^(21)^. Além de uma tarefa de processamento como resolução de problemas (por exemplo, subtração de 3 por 3), a atenção e a demanda visual durante a caminhada (como carregar um copo enquanto caminha) podem prejudicar o desempenho da marcha, o funcionamento cognitivo ou ambos^(22)^.

Os achados deste estudo mostram que o declínio cognitivo não foi relacionado ao pior desempenho na velocidade da marcha ou em tarefas duplas, tanto motoras quanto cognitivas. No entanto, constatou-se que quanto maior o escore cognitivo dos idosos, melhor o desempenho no tempo de marcha avaliado.

Analisar a marcha como uma tarefa complexa é benéfico para a saúde do idoso, pois pode aumentar sua validade como marcador para identificação de declínio cognitivo precoce^(23)^. A velocidade da marcha pode ser utilizada como um índice da função cognitiva e do estado de saúde atual do indivíduo^(6)^.

O desenvolvimento de instrumentos para avaliar simultaneamente déficits relacionados à fragilidade física e cognitiva é importante pela associação entre a síndrome da fragilidade e a cognição em idosos. Apesar disso, pouco se sabe sobre o quanto esses aspectos interferem no desempenho de testes de tarefa dupla^(23)^. O referido estudo ainda verificou influência da síndrome da fragilidade e da atividade física e cognição mensuradas no TUG e TUG-DT, tendo sido também comparados os desempenhos de ambos os testes entre idosos frágeis. Os resultados mostraram ausência de diferenças na cognição entre os grupos e que o grupo não frágil, quando comparado ao frágil, exigiu menos tempo para realizar o TUG. Em relação ao TUG-DT, a cognição e a idade influenciaram o tempo gasto para realizar a tarefa, mas não foram encontradas diferenças entre os grupos. Assim, idosos frágeis tiveram pior desempenho no TUG quando comparados aos participantes não frágeis, e o teste de dupla tarefa não diferenciou idosos frágeis de não frágeis, independentemente do desempenho cognitivo^(23)^.

As alterações relacionadas à dupla tarefa na velocidade da marcha são sensíveis e diferenciam pessoas saudáveis daquelas com comprometimento cognitivo. A velocidade da marcha é mais lenta em pessoas mais velhas e menos educadas^(6, 23)^. Ser mulher, solteiro e ter baixa renda são características do grupo de ritmo mais lento; também neste grupo, a pontuação da função cognitiva foi menor. No mesmo estudo, os autores também encontraram correlação entre velocidade de marcha mais lenta e diminuição do funcionamento cognitivo e sugeriram que a reabilitação geriátrica deve incluir não apenas o treinamento da marcha e o fortalecimento dos músculos das pernas, mas também o treinamento das habilidades cognitivas frontais e gerais para melhor desempenho da marcha^(6, 22, 23)^.

A realização de tarefas secundárias durante a caminhada diminui a velocidade da marcha, em um sinal de que a marcha depende do funcionamento cognitivo^(5, 9)^. No entanto, também é possível que o declínio na velocidade da marcha decorra do estado de fragilidade da população idosa^(6)^.

A relação entre cognição e marcha durante a caminhada de dupla tarefa pode ser uma ferramenta para monitorar a deterioração da marcha ao longo do envelhecimento, mesmo as leves. É claro que um desafio cognitivo durante a execução de uma tarefa motora interfere em seu desempenho. Em geral, a capacidade de lidar com uma tarefa dupla diminui progressivamente com o envelhecimento, embora as razões para isso permaneçam obscuras^(9)^. O presente estudo mostrou que a presença de hipertensão arterial influenciou a piora do desempenho da marcha dos participantes. Por outro lado, a velocidade da marcha é considerada um preditor precoce de doença cardiovascular^(6)^. Estudo conduzido em Taiwan para investigar a associação entre a velocidade da caminhada e o risco de doença cardiovascular entre populações de meia-idade e idosos na comunidade assinalou associação entre velocidade lenta da marcha e risco aumentado de doença cardiovascular em pessoas de meia-idade, porém não em idosos^(6)^. Cabe ressaltar que o desempenho da velocidade da marcha foi pior em idosos com idade mais avançada e menor escolaridade, com menores escores cognitivos e com hipertensão arterial, demonstrado em estudo recente^(6)^.

O presente estudo apresenta algumas limitações. Há diferenças na padronização da avaliação da marcha e da avaliação cognitiva descritas na literatura. Em relação à marcha, os estudos que a avaliaram em idosos por meio do TUG não seguiram a distância específica, tal como feito neste estudo, o que dificulta a comparação de resultados e a análise de populações. O mesmo acontece com a avaliação cognitiva, pois há vários instrumentos para avaliar essa variável e é difícil comparar o desempenho em diferentes medidas. Apesar dessas limitações, os estudos devem ser incentivados a utilizar distintos instrumentos de avaliação, se necessário, para fornecer confiabilidade e reprodutibilidade para as avaliações em unidades de saúde.

Como o objetivo de detectar déficits precocemente para identificar necessidades, organizar, implementar e reavaliar a eficiência das intervenções, a fim de promover melhorias na qualidade de vida dos idosos, é fundamental e integra as habilidades e competências dos profissionais de saúde, o presente estudo contribuirá para qualificar a implementação de intervenções direcionadas para melhorar a marcha e/ou manter a cognição.

## Conclusão

Com base no objetivo proposto, por meio da análise da influência da fragilidade e do declínio cognitivo no desempenho de dupla tarefa em idosos, este estudo demonstrou que a fragilidade é um fator com maior interferência no desempenho de dupla tarefa em idosos quando comparada ao desempenho cognitivo. Importante mencionar que a forte associação entre marcha e cognição tem sido descrita em muitos estudos. Vale ressaltar também que idosos com déficits de marcha têm maior risco de desenvolver déficits cognitivos, os quais, por sua vez, estão associados à piora da marcha.

# Translation: Influencia de la fragilidad y disminución cognitiva en la doble tarea de adultos mayores: un estudio transversal analítico*

**DOI:** 10.1590/1518-8345.7159.4484

**Authors:** Francine Golghetto Casemiro, Lucas Pelegrini Nogueira de Carvalho, Fernanda de Brito Matiello, Marcela Cristina Resende, Rosalina Aparecida Partezani Rodrigues

**Affiliations:** 1 Universidade de São Paulo, Escola de Enfermagem de Ribeirão Preto, Centro Colaborador de la OPS/OMS para el Desarrollo de la Investigación en Enfermería, Ribeirão Preto, SP, Brasil; 2 Universidade Federal de São Carlos, São Carlos, SP, Brasil; 3 Becaria de la Coordenação de Aperfeiçoamento de Pessoal de Nível Superior (CAPES), Brasil; 4 Hospital das Clínicas da Faculdade de Medicina de Ribeirão Preto, Ribeirão Preto, SP, Brasil; 5 Becaria del Conselho Nacional de Desenvolvimento Científico e Tecnológico (CNPq), Brasil

**Keywords:** Adulto Mayor, Fragilidad, Disfunción Cognitiva, Geriatría, Comportamiento Multifuncional, Cognición

## Abstract

**Objetivo:**

analizar la influencia de la fragilidad y disminución cognitiva en el desempeño de la doble tarea en adultos mayores.

**Método:**

estudio transversal realizado en adultos mayores, en un ambulatorio de geriatría, en Sao Paulo. Fueron utilizados datos sociodemográficos, el desempeño cognitivo, el fenotipo de fragilidad, la velocidad de marcha y la doble tarea. El análisis fue descriptivo y el modelo de regresión linear múltiple.

**Resultados:**

participaron 219 adultos mayores, con media de edad de 72,55 años, intervalo etario entre 60 y 79 años (82,65%), sexo femenino (70,32%) y 86 (39,27%) adultos mayores frágiles; 123 (57,48%) presentaron disminución cognitiva. El tiempo medio de caminada simple fue de 15,95 (7,02%) segundos; en la doble tarea motora fue 17,64 (8,44%) y en la doble tarea cognitiva, 23,88 (11,87%). Mujeres mayores sin compañero, viviendo con la familia y con baja escolaridad (0-4 años) necesitaron de más tiempo para realizar la doble tarea cognitiva y motora en comparación con en el tiempo para caminada simple. Hombres mayores frágiles presentaron empeoramiento en el desempeño de la marcha, tanto en las tareas motoras/cognitivas simples como en las dobles.

**Conclusión:**

adultos mayores frágiles presentaron empeoramiento significativo en el desempeño en la marcha simple y en las tareas motoras/cognitivas dobles y aquellas con y sin disminución cognitiva no se diferenciaron. Intervenciones multidisciplinares deben tener como objetivo promover la salud del adulto mayor.

## Destacados

**(1)** La fragilidad y la sarcopenia son síndromes importantes que deben ser evaluados en los adultos mayores.**(2)** Los adultos mayores con DM2 tienen mayor vulnerabilidad a desarrollar fragilidad. **(3)** Las enfermeras deben implementar medidas preventivas para abordar la fragilidad y la sarcopenia.

## Introducción

La proporción de adultos mayores aumenta mundialmente cuando comparada con otros intervalos etarios^(1)^. El proceso de envejecimiento y la manifestación de morbilidades, cuando asociados a la incapacidad, pueden influenciar el proceso de fragilidad en la población de adultos mayores^(2)^. La fragilidad es una condición clínica que lleva al aumento de la vulnerabilidad y está asociada a resultados negativos como caídas, incapacidad, hospitalización y mortalidad^(3)^.

Una de las dimensiones para evaluar el fenotipo de fragilidad es la velocidad de marcha^(2)^, ella es afectada directamente por el proceso de envejecimiento^(4)^. A medida que las personas envejecen, aparece una disminución de 20% en la velocidad de marcha por década; esa reducción interfiere en la independencia, lo que, a su vez, perjudica el desempeño de las Actividades de la Vida Diaria (AVDs)^(4)^. Los disturbios de la marcha son prevalentes en adultos mayores y pueden ocasionar caídas, inmovilidad, institucionalización y aumento de la mortalidad^(5)^. El comprometimiento de la marcha puede preceder al comprometimiento cognitivo y a la dificultad en las AVDs; además, representa una etapa inicial del proceso de incapacidad^(5)^. Sin embargo, la relación entre marcha y cognición todavía necesita de más investigación^(6)^.

En adultos mayores con más edad, la velocidad más lenta de la marcha está asociada al riesgo de demencia y disminución cognitiva^(6)^. Además de eso, la velocidad de marcha puede diferenciar adultos mayores saludables de aquellos con disminución cognitiva^(7)^. La literatura sugiere la existencia de una correlación entre el bajo desempeño cognitivo y la condición de fragilidad en adultos mayores^(8)^. Por otro lado, un metaanálisis identificó que la asociación entre la disminución física-funcional y la disminución neurológica cognitiva, ha sido cada vez más recurrente; también, mostró que el empeoramiento en el desempeño cognitivo ocurre durante la transición de la condición prefrágil para la condición frágil, lo que caracteriza un fenotipo de adulto mayor “cognitiva y físicamente frágil”^(7)^. Cognición y fragilidad son potenciales predictores de la mortalidad precoz, en la población de adultos mayores^(8)^. Finalmente, la velocidad de la marcha también puede diferenciar los adultos mayores en lo que se refiere a su estado de fragilidad (o sea: frágil, prefrágil y no frágil) cuando hay reducción en la velocidad de marcha durante la doble tarea^(9)^.

A pesar de que las alteraciones en la marcha causadas por la doble tarea son observadas en adultos saludables, esa condición aumenta con la edad y en personas con disminución cognitiva^(10)^. La intensidad de esa alteración está directamente relacionada con la disminución cognitiva^(10)^; eso se debe a la reducción de la atención, lo que compromete la estabilidad de la marcha^(11)^.

La asociación de la marcha tanto en la caminada simple como en la caminada de doble tarea, puede ayudar a entender la deterioración de la marcha en el envejecimiento y el vínculo entre la función motora y cognitiva^(11)^. La caminada de doble tarea también puede ser útil para monitoreo de deterioraciones sutiles y diversas de la marcha en el envejecimiento^(9)^; entender esas relaciones contribuirá para la planificación de intervenciones capaces de mantener o recuperar estándares adecuados de marcha en adultos mayores. Por lo tanto, el objetivo de este estudio es analizar la influencia de la fragilidad y de la disminución cognitiva en el desempeño de la doble tarea, en adultos mayores.

Entonces, el objetivo es analizar la influencia de la fragilidad y de la disminución cognitiva en el desempeño de la doble tarea, en adultos mayores que viven en la comunidad.

## Método

### Diseño, período y local del estudio

Este es un estudio analítico, observacional y transversal, conducido de acuerdo con las directrices del *Strengthening the Reporting of Observational Studies in Epidemiology* (STROBE)^(12)^. El protocolo de evaluación fue desarrollado entre agosto y septiembre de 2019. El entrenamiento del equipo ocurrió en octubre de 2019. La recolección de datos comenzó en noviembre de 2019 y continuó hasta febrero de 2020, en el Centro de Salud Escuela de la Facultad de Medicina de la Universidad de Sao Paulo, en Ribeirao Preto, estado de Sao Paulo.

### Población y muestra

Para calcular el tamaño de la muestra, un coeficiente de determinación R-al cuadrado de 0,10 fue considerado en un modelo de regresión linear múltiple con los predictores. El nivel de significación o tasa de error Tipo I fue definido en α = 0,01; la tasa de error Tipo II en β = 0,1; lo que resultó en un poder estadístico predeterminado de 90%. Utilizando el aplicativo PASS (*Power Analysis and Sample Size*), versión 13, e introduciendo los valores anteriormente citados, se obtuvo un tamaño de muestreo mínimo de n=206. Entre tanto, considerando una pérdida de la muestra de 20% (negativas de participación), el número final de intentos de entrevistas fue de n=258. La principal variable dependiente fue el tiempo registrado para la ejecución del test de marcha de doble tarea motora-cognitiva.

Los participantes fueron contactados antes de la consulta médica y aquellos que aceptaron participar fueron evaluados después de la consulta médica. Las entrevistas fueron realizadas por alumnos de graduación, postgraduación y postdoctorado del Núcleo de Investigación en Geriatría y Gerontología (NUPEGG), de la Escuela de Enfermería de Ribeirão Preto-USP. Vale destacar que los entrevistadores fueron previamente entrenados por el líder del grupo, con el objetivo de estandarizar las técnicas de evaluación.

### Criterios de inclusión y exclusión

La población del estudio estuvo compuesta por adultos mayores comunitarios, usuarios del ambulatorio de una Unidad de Salud Pública. Para participar del estudio, fue necesario atender a los siguientes criterios de inclusión: tener edad igual o superior a 60 años; pertenece a uno de ambos sexos; residir en domicilio, en el municipio de Ribeirão Preto; ser capaz de comunicarse verbalmente; y, no tener diagnóstico de demencia.

### Instrumentos de evaluación

- *Cuestionario sociodemográfico:* Instrumento elaborado por el NUPEGG en 2006, que contempla informaciones sobre sexo (masculino y femenino); edad (en años); estado civil (soltero, casado, separado, divorciado o viudo); renta del adulto mayor (renta líquida en reales); escolaridad (en años de estudio formal); número de hijos; número de personas que viven en el mismo domicilio; y, situación de jubilación (si o no).
- *Desempe* **ñ** *o cognitivo:* para evaluar esa variable, fue utilizado el Mini-Examen del Estado Mental (MEEM); este fue traducido y validado para su versión en portugués brasileño; las notas de corte variaron de acuerdo con el nivel educacional del participante. El MEEM también fue validado para ambientes hospitalarios/clínicos y estudios poblacionales^(13)^. Sus puntajes variaron de cero a 30; los puntos de corte sugeridos por los autores fueron los siguientes: 20 para analfabetos, 24 para aquellos con 1 a 4 años de escolaridad, 26,5 puntos para aquellos con 5 a 8 años de escolaridad, 28 puntos para aquellos entre 9 y 11 años; y, 29 puntos para aquellos con más de 11 años de escolaridad^(13)^.
- *Fenotipo de fragilidad:* para esta evaluación, los participantes fueron conducidos a una sala adaptada con sillas, balanza, estadiómetro y dinamómetro hidráulico manual marca JAMAR. De acuerdo con el fenotipo de fragilidad^(2)^ fueron evaluadas las siguientes medidas: peso corporal (obtenido por balanza); fatiga; prensión manual; velocidad de marcha; y, nivel de actividad física. La fatiga fue evaluada por dos preguntas de una escala de clasificación de depresión. Fue positivo cuando los participantes declararon la necesidad de hacer mucho esfuerzo para lidiar con las tareas o que no conseguían realizar sus tareas habituales, en tres días o más en la semana. La fuerza de prensión manual fue medida usando un dinamómetro hidráulico portátil en la mano dominante. Los resultados fueron ajustados para género e índice de masa corporal (IMC). Para la velocidad de la marcha, fue calculado el tiempo medio que los participantes utilizaron para recorrer una distancia de 4,6 metros. Ajustes de acuerdo con sexo y altura también fueron aplicados; el tiempo medio fue calculado con base en la velocidad en tres intentos. Por último, el nivel de actividad física fue evaluado por el Cuestionario Internacional de Actividad Física (IPAQ), propuesto por la Organización Mundial de la Salud (OMS) en 1998. Este instrumento fue previamente validado en varios países, incluyendo Brasil^(14)^, el que ha sido ampliamente utilizado en todo el mundo^(15)^.
- *Velocidad de marcha:* fue realizado el test *Timed Up and Go* (TUG); los participantes fueron instruidos a: levantarse de una silla de 45 cm de altura con brazos; caminar una distancia de tres metros en su velocidad de marcha habitual; darse vuelta, retornar a la silla y sentarse. Para tener un buen desempeño, los adultos mayores necesitan de movilidad, agilidad y tener sus funciones ejecutivas en buenas condiciones; por tanto el TUG no es un test simple de caminada^(15)^. El test se inicia con el comando “va” y termina cuando el adulto mayor se sienta nuevamente. Para esa evaluación, el tiempo de ejecución del recorrido, durante los tres metros, fue registrado con validez en Brasil; este ha sido ampliamente utilizado en el mundo^(14)^.
- *Doble tarea*: El TUG puede ser aplicado rápidamente, es fácil de ser reproducido y también ha sido asociado a tareas secundarias motoras y/o cognitivas (por ejemplo, *Dual-task* TUG)^(16)^. El *Dual-Task* TUG (DT-TUG) es un biomarcador confiable para la disminución motora en la población de adultos mayores, ya que identifica alteraciones en los parámetros de marcha y auxilia en el diagnóstico diferencial de la Enfermedad de Alzheimer^(16)^. En este estudio, la tarea doble cognitiva (Cog-DT) fue realizada cuando el adulto mayor caminando recorría los tres metros y contaba números de 3 en 3 (comenzando con un número entre 80 y 99). Para la tarea doble motora (Mot-DT), fue establecido lo siguiente: el adulto mayor debe caminar una distancia de 3 metros sosteniendo un vaso de agua. Las medias de los grupos fueron comparadas; es decir no fue utilizado un instrumento de validación, ya que las medias de los participantes fueron comparadas de acuerdo con los grupos de clasificación referentes a la fragilidad.

### Análisis de los datos

La variable dependiente en este estudio fue el tiempo necesario para completar la tarea doble (Mot-DT). Las variables independientes incluyen factores sociodemográficos/clínicos, la función cognitiva, la fragilidad y la velocidad de marcha.

Todas las variables fueron sometidas al análisis estadístico. Para variables cualitativas fueron utilizadas medidas de frecuencia absoluta; para las variables categóricas medidas relativas; y, para las variables cuantitativas se utilizaron medidas de tendencia central (media, mediana, mínimo y máximo) y de variabilidad (amplitud y desviación estándar). Para relacionar la variable de resultado con las variables exploratorias, fue utilizada la regresión linear y el test de correlación de Pearson. Cuando los prerrequisitos para el uso de pruebas paramétricas no fueron atendidos, fue aplicado un test no paramétrico (Test de Mann-Whitney). Todas las pruebas adoptaron un nivel de significación de *p* ≤0,05.

Para analizar la influencia de las características sociodemográficas, clínicas, de fragilidad y disminución cognitiva, en el desempeño del Mot-DT, fue realizado un análisis bivariado incluyendo el test *t* de Student, para predictores dicotómicos; así como correlaciones de Pearson para predictores cuantitativos. El análisis de la influencia simultánea de las características sociodemográficas, clínicas, de fragilidad y los predictores de disminución cognitiva en la doble tarea (Mot-DT) y de velocidad de marcha, incluyen el análisis de regresión linear múltiple.

Para analizar la influencia de las características sociodemográficas y clínicas, de fragilidad y de las variables de disminución cognitiva, en la velocidad de marcha, fue utilizada la regresión linear múltiple. Para el análisis de la influencia de las características sociodemográficas y clínicas, fragilidad y disminución cognitiva en el desempeño del Cog-DT, fueron utilizados análisis bivariados, incluyendo el test *t* de Student, para predictores dicotómicos, así como correlaciones de Pearson para predictores cuantitativos. La evaluación - de la influencia simultánea de características sociodemográficas y clínicas, de fragilidad y predictores de disminución cognitiva en la doble tarea (cognitiva) y velocidad de marcha - incluyó el análisis de regresión linear múltiple. Para realizar la regresión linear simple y múltiple, realizamos una búsqueda en la literatura con base en nuestros datos; por tanto, las variables de interés fueron: edad, educación, género, artritis, hipertensión arterial y fragilidad. El SAS *System for Windows (Statistical Analysis System)*, versión 9.2 (SAS *Institute Inc.*, 2002-2008, Cary, NC, EUA), fue utilizado para ejecutar análisis estadísticos.

### Aspectos éticos

La Secretaría de la Salud de Ribeirão Preto autorizó la investigación y recibió la aprobación número 5.427.143 del Comité de Ética e Investigación (CEI) de la Escuela de Enfermería de Ribeirão Preto de la Universidad de Sao Paulo, conforme Resolución 466/2012 del Consejo Nacional de Salud. Los participantes fueron informados sobre la investigación; la evaluación se inició solamente después de la firma del Término de Consentimiento Libre e Informado. Este manuscrito hace parte de un proyecto mayor de uno de los autores, titulado “Biomarcadores relacionados a la fragilidad y sarcopenia en adultos mayores”.

## Resultados

Los participantes (n=219) eran mayoritariamente blancos (69,41%), del sexo femenino (70,32%), con media de edad de 72,55 (DE=7,3) años; el intervalo etario predominante fue entre 60 y 79 años (82,55%). Del total de la muestra, 52,05% tenían compañero y la media de hijos fue de 3,04. Es importante destacar que 97,72% eran usuarios del Sistema Único de Salud. La escolaridad varió de cero a más de 12 años, con media de 5,15 años de estudio (DE=3,82).

En relación a la renta, la media mensual fue de R$ 1.459,60 reales (DE=934,55); la mayoría de los participantes (n=155, 70,78%) recibía un valor mayor que un salario mínimo. En relación a la fuente de renta, 174 adultos mayores (79,82%) no poseían salario de jubilación y 179 participantes recibían pensión (82,11%). Un total de 202 adultos mayores todavía estaban trabajando (94,04%) y 215 refirieron recibir donaciones (98,62%). Además de eso, 94,95% de los participantes vivían en casas arrendadas.

Las características clínicas también fueron evaluadas. La mayoría de los participantes era: prefrágil (n=131, 59,82%); con disminución cognitiva (n=123, 57,48%); refirió buena memoria (n=114, 52,05) y clasificó su memoria como buena hace un año (n=144, 65,75%). La velocidad media de marcha de los adultos mayores fue de 15,95 segundos (DE=7,02) y la velocidad media de la marcha para Mot-DT y Cog-DT fueron, respectivamente, 17,64 (DE=8,44) y 23,88 (DE=11,87). La Tabla 1 muestra las características sociodemográficas y clínicas de los participantes.

**Tabla 1 -:** Características sociodemográficas y clínicas de los adultos mayores residentes en la comunidad (n = 219). Ribeirão Preto, SP, Brasil, 2022

**Variable**	**Categoría**	n*	**%** ^†^
Género	Femenino	154	70,32
	Masculino	65	29,68
Edad	Media (=DE)^‡^	72,55 (7,3)	-
	A. mayor más joven 60-79	181	82,65
	A. mayor más viejo >80	38	17,35
Etnia	Blanco	155	69,41
	Pardo	38	17,35
	Negro	28	12,79
	Desconocido	1	0,46
Vivir con un compañero	No	105	47,95
	Si	114	52,05
Servicios de salud utilizados	SUS	214	97,72
	Privado	4	1,83
	Farmacia	1	0,46
Estado civil	Soltero	24	10,96
	Casado	114	52,05
	Divorciado	12	5,48
	Separado	8	3,65
	Viudo	60	27,40
	Otro	1	-
Escolaridad	Media (=DE)^‡^	5,15 (3,82)	-
	0 – 4	131	61,21
	5 – 8	52	24,30
	09-11	14	6,54
	12 o más	17	7,94
Salario de jubilación	Si	44	20,18
	No	174	79,82
Pensión	Si	179	82,11
	No	39	17,89
Estado de trabajo	Activo	202	94,04
	Inactivo	13	5,96
Renta mensual	R$1.039,00^ǁ^	58	26,48
	Más de un salario mínimo	155	70,78
Recibe alguna donación	Si	215	98,62
	No	3	1,38
Vive en una casa arrenda	Si	207	94,95
	No	11	5,05
Número de hijos	Media (=DE)^‡^	3.04 (2.12)	-
Viviendo con	Solo	32	14,61
	Con un compañero	66	30,13
	Otro	119	54,33
Fragilidad	No Frágil	2	0,91
	Prefrágil	131	59,82
	Frágil	86	39,27
Disminución cognitiva	Sin	91	42,52
	Con	123	57,48
Autoevaluación de memoria	Excelente	10	4,57
	Muy Buena	24	10,96
	Buena	114	52,05
	Regular	58	26,48
	Mala	12	5,48
	Muy mala	1	0,46
Memoria en comparación a un año atrás	Buena	21	9,59
	Igual	144	65,75
	Peor	54	24,66
Variable		Media (DE)^‡^	
Velocidad de marcha		15.95 (7.02)	
DT-Mot (segundos)^§^		17.64 (8.44)	
DT-Cog (segundos)^¶^		23.88 (11.87)	

*n = Número de participantes; ^†^%= Frecuencia; ^‡^DE = Desviación estándar; ^§^Mot-DT = Doble tarea motora; ^||^Salario mínimo del país de la investigación; ^¶^Cog-DT = Doble tarea cognitiva

Los resultados demuestran que la presencia de la disminución cognitiva no está relacionada a: la velocidad de la marcha (p=0,095), la doble tarea motora (p=0,124) y a la doble tarea cognitiva (p=0,069). La fragilidad, por otro lado, fue una variable importante en relación al desempeño de la marcha, tanto en la marcha simple (p<0,001) como en la doble tarea (p<0,001). Así, de acuerdo con los análisis, se puede afirmar que la fragilidad tiene mayor impacto en el desempeño de la doble tarea y en la velocidad de la marcha simple, cuando comparada con la disminución cognitiva (Tabla 2).

Las Tabla 3 y Tabla 4 presentan los resultados de los análisis de regresión linear simple y múltiple (con criterio *Stepwise* para selección de variables), respectivamente, para estudiar la relación entre las variables de interés y la velocidad de marcha.

**Tabla 2 -:** Comparación de la velocidad de la marcha, Mot-DT* y Cog-DT^†^ entre las variables categóricas disminución cognitiva y fragilidad (n = 219). Ribeirão Preto, SP, Brasil, 2022

Comparación con Disminución Cognitiva (MMSE)^‡^									
	Variable		N^§^	Media	DE^||^	Mediana	Z^¶^	P**	
Sin disminución		Velocidad de Marcha	91	15,02	5,69	13,50	1,67	p=0,095	
		DT-Mot*	91	16,57	6,41	15,00	1,54	p=0,124	
		DT-Cog^†^	91	22,42	9,40	20,00	1,82	p=0,069	
Con disminución		Velocidad de Marcha	123	16,69	7,93	14,50			
		DT-Mot*	123	18,51	9,75	16,70			
		DT-Cog^†^	123	25,15	13,47	22,00			
**Comparación con Fragilidad**									
	Variable		N^§^	Media	DE^||^	Mediana	Z^¶^	p**	Comparación
No Frágil	Velocidad de Marcha		2	10,00	0,71	10,00	16,34	p<0,001	1≠3
(1)	Mot-DT*		2	12,00	1,41	12,00	26,81	p<0,001	1≠3
	Cog-DT^†^		2	18,50	6,36	18,50	15,43	p<0,001	1≠3
	Variable		N^§^	Media	DE^||^	Mediana	Z^¶^	p**	Comparación
Prefrágil	Velocidad de Marcha		131	14,84	5,86	13,50			
(2)	Mot-DT*		131	15,83	6,25	15,00			
	Cog-DT^†^		131	21,62	8,30	20,00			
Frágil	Velocidad de Marcha		86	17,78	8,23	15,75			
(3)	Mot-DT*		86	20,53	10,43	18,00			
	Cog-DT^†^		86	27,45	15,29	23,50			
**Comparación con Fragilidad (Agrupada)**									
	Variable		N^§^	Media	DE^||^	Mediana	Z^¶^	p^††^	
No Frágil/Prefrágil	Velocidad de Marcha		133	14,76	5,85	13,00	3,69	p<0,001	
	DT-Mot*		133	15,77	6,22	15,00	5,07	p<0,001	
	DT-Cog^†^		133	21,57	8,26	20,00	3,90	p<0,001	
Frágil	Velocidad de Marcha		86	17,78	8,23	15,75			
	DT-Mot*		86	20,53	10,43	18,00			
	DT-Cog^†^		86	27,45	15,29	23,50			

*DT-Mot = Doble Tarea motora; ^†^DT-Cog = Doble Tarea Cognitiva; ^‡^MMSE = Mini Examen del Estado Mental; ^§^n = Número de participantes; ^||^DE = Desviación Estándar; ^¶^Z = Estadística Z del test; **Valor de *p* referente al test de Mann-Whitney para comparación de variables entre 2 grupos; ^††^Valor de *p* referente al test de Kruskal-Wallis para comparación de variables entre 3 o más grupos

**Tabla 3 -:** Análisis de regresión linear simple para velocidad de marcha (n = 219). Ribeirão Preto, SP, Brasil, 2022

Velocidad de Marcha				
Variable	Categorías	Beta* (se)^†^	p^‡^	R2^§^
Género	Masculino (ref.)^ǁ^	-	-	-
	Femenino	30,87 (9,15)	<0,001	0,0498
Grupo etario	60-79 años (ref.)^ǁ^	-	-	-
	≥80 años	47,95 (10,85)	<0,001	0,0826
Edad (en **año**s)	-	0,29 (0,06)	<0,001	0,0851
Años de Educación	0-4 años (ref.)^ǁ^	-	-	-
	5-8 años	-24,15 (10,05)	0,017	-
	9-11 años	-51,85 (17,24)	0,003	-
	≥12 años	-53,11 (15,80)	<0,001	0,0889
Fragilidad	No Frágil/Prefrágil (ref.)^ǁ^	-	-	-
	Frágil	32,30 (8,50)	<0,001	0,0624
Disminución Cognitiva	No (ref.)^ǁ^	-	-	-
	Si	14,80 (8,78)	0,093	0,0132
Hipertensión Arterial	No (ref.)^ǁ^	-	-	-
	Si	34,11 (8,55)	<0,001	0,0683
Artritis	No (ref.)^ǁ^	-	-	-
	Si	24,75 (8,44)	0,004	0,0382
**Tarea Motora Doble**				
Variable	Categorías	Beta* (se)^†^	p^‡^	R2^§^
G**é**nero	Masculino (ref.)^ǁ^	-	-	-
	Femenino	20,14 (9,27)	0,031	0,0213
Grupo etario	60-79 años (ref.)^ǁ^	-	-	-
	≥80 años	61,55 (10,51)	<0,001	0,1365
Variable	Categorías	Beta* (se)^†^	p^‡^	R2^§^
Edad (en años)	-	0,37 (0,06)	<0,001	0,1341
Años de Educación	0-4 años (ref.)^ǁ^	-	-	-
	5-8 años	-28,83 (9,75)	0,004	-
	9-11 años	-67,05 (16,73)	<0,001	-
	≥12 años	-65,94 (15,34)	<0,001	0,1399
Fragilidad	No Frágil/Prefrágil (ref.)^ǁ^	-	-	-
	Frágil	44,34 (8,24)	<0,001	0,1178
Disminución Cognitiva	No (ref.)^ǁ^	-	-	-
	Si	13,64 (8,78)	0,122	0,0113
Hipertensión Arterial	No (ref.)^ǁ^	-	-	-
	Si	35,23 (8,52)	<0,001	0,0730
Artritis	No (ref.)^ǁ^	-	-	-
	Si	14,78 (8,53)	0,084	0,0136
**Tarea Cognitiva Doble**				
Variable	Categorías	Beta* (se)^†^	p^‡^	R2^§^
Género	Masculino (ref.)^ǁ^	-	-	-
	Femenino	28,84 (9,17)	0,002	0,0435
Grupo etario	60-79 años (ref.)^ǁ^	-	-	-
	≥80 años	43,38 (10,93)	<0,001	0,0677
Edad (en años)	-	0,30 (0,06)	<0,001	0,0872
Años de Educación	0-4 años (ref.)^ǁ^	-	-	-
	5-8 años	-37,49 (9,58)	<0,001	-
	9-11 años	-75,02 (16,44)	<0,001	-
	≥12 años	-60,43 (15,07)	<0,001	0,1614
Fragilidad	No Frágil/Prefrágil (ref.)^ǁ^	-	-	-
	Frágil	34,17 (8,46)	<0,001	0,0699
Disminución Cognitiva	No (ref.)^ǁ^	-	-	-
	Si	15,89 (8,72)	0,070	0,0154
Hipertensión Arterial	No (ref.)^ǁ^	-	-	-
	Si	28,06 (8,65)	0,001	0,0463
Artritis	No (ref.)^ǁ^	-	-	-
	Si	14,19 (8,54)	0,098	0,0125

*Beta = Valor estimado o coeficiente angular (inclinación) en la recta de regresión; ^†^se = Error estándar de beta; ^‡^ *p* = Valor; ^§^R2: coeficiente de determinación (% variabilidad de la variable respuesta, explicada por la variable independiente); ^ǁ^Ref = Referencia. Variables sin distribución normal fueron transformadas en *ranks*

**Tabla 4 -:** Análisis de regresión linear múltiple para la velocidad de marcha (n = 219). Ribeirão Preto, SP, Brasil, 2022

Velocidad de Marcha				
Variables Seleccionadas	Categorías	Beta* (se)^†^	p^‡^	R2^§^
Grupo Etario	60-79 años (ref.)^§^	-	-	-
	≥80 años	41,56 (10,22)	<0,001	0,0845
Hipertensión Arterial	No (ref.)^ǁ^	-	-	-
	Si	26,27 (7,99)	0,001	0,0709
Género	Masculino (ref.)^ǁ^	-	-	-
	Femenino	25,02 (8,71)	0,005	0,0473
Variables Seleccionadas	Categorías	Beta* (se)^†^	p^‡^	R2^§^
Fragilidad	No Frágil/Prefrágil (ref.)^ǁ^	-	-	-
	Frágil	20,11 (8,01)	0,013	0,0356
Años de Educación	0-4 años (ref.)^ǁ^	-	-	-
	5-8 años	-13,66 (9,19)	0,139	-
	9-11 años	-26,02 (15,84)	0,102	-
	≥12 años	-41,92 (14,30)	0,004	0,0367
Artritis	No (ref.)^ǁ^	-	-	-
	Si	17,14 (7,97)	0,033	0,0160
**Tarea Dupla Motora**				
Grupo Etario	60-79 años (ref.)^ǁ^	-	-	-
	≥80 años	48,62 (9,58)	<0,001	0,1376
Fragilidad	No Frágil/Prefrágil (ref.)^ǁ^	-	-	-
	Frágil	30,84 (7,49)	<0,001	0,0851
Hipertensión Arterial	No (ref.)^ǁ^	-	-	-
	Si	28,52 (7,48)	<0,001	0,0561
Años de Educación	0-4 años (ref.)^ǁ^	-	-	-
	5-8 años	-14,49 (8,64)	0,095	-
	9-11 años	-38,22 (14,90)	0,011	-
	≥12 años	-52,75 (13,45)	<0,001	0,0689
Género	Masculino (ref.)^ǁ^	-	-	-
	Femenino	20,16 (7,86)	0,011	0,0202
**Tarea Dupla Cognitiva**				
Fragilidad	No Frágil/Prefrágil (ref.)^ǁ^	-	-	-
	Frágil	23,32 (7,92)	0,004	0,0734
Años de Educación	0-4 años (ref.)^ǁ^	-	-	-
	5-8 años	-28,24 (9,13)	0,002	-
	9-11 años	-54,21 (15,75)	<0,001	-
	≥12 años	-50,63 (14,22)	<0,001	0,1322
Género	Masculino (ref.)^ǁ^	-	-	-
	Femenino	25,44 (8,31)	0,003	0,0337
Grupo Etario	60-79 años (ref.)^ǁ^	-	-	-
	≥80 años	28,45 (10,13)	0,006	0,0255
Hipertensión Arterial	No (ref.)^ǁ^	-	-	-
	Si	19,81 (7,90)	0,013	0,0218

*Beta = Valor estimado o coeficiente angular (inclinación) en la recta de regresión; ^†^se = Error estándar de beta; ^‡^ *p* = Valor; ^§^R2: coeficiente de determinación; ^ǁ^Ref= Referencia. Criterio *stepwise* para selección de variables. R2 total: 0,2909. Intercepto (se): 60,70 (10,00); p<0,001. Variables sin distribución normal fueron transformadas en *ranks*

El análisis de regresión linear simple mostró que ser del sexo femenino, tener 80 años o más (p<0,001), tener entre 0 t 4 años de estudio (p<0,001), ser frágil (<0,001), tener hipertensión arterial (<0,001) y tener artritis (p<0,004), son factores que empeoran el desempeño de la velocidad de marcha en adultos mayores. Los adultos mayores con mayor valor para la velocidad de marcha fueron: aquellos con edad ≥80 años, del sexo femenino, escolaridad entre 0 y 4 años, con hipertensión arterial, con artritis y fragilidad.

En relación al Mot-DT, el análisis de regresión linear simple mostró que hubo relación entre: tener 80 años o más (p<0,001); tener entre 0 y 4 años de estudio (p<0,001); ser frágil (p<0,001); y, tener hipertensión arterial (p<0,001), con peor desempeño en la tarea motora dupla. Por otro lado, el análisis de regresión linear simple indica que adultos mayores con 80 años o más (p<0,001), con 0 a 4 años de estudio (p<0,001), frágiles (p<0,001) y con hipertensión arterial (p=0,001), son personas con peor desempeño en la doble tarea cognitiva. Hubo relación significativa entre la edad (p<0,001), género (p=0,005), escolaridad (p=0,004), hipertensión arterial (p=0,001), artritis (p=0,033) y fragilidad (p=0,013) con la velocidad de marcha.

Con base en los resultados del análisis múltiple, hubo relación significativa entre edad (p<0,001), escolaridad (p<0,001), género (p=0,011), fragilidad (p<0,001), hipertensión arterial (p<0,001) y doble tarea motora. Los adultos mayores con mayor tiempo para realizar el Mot-DT fueron los: con edad ≥80 años, con 0-4 años de estudio, del sexo femenino, frágiles y con hipertensión arterial.

En el análisis múltiple hubo relación significativa entre fragilidad (p=0,004), escolaridad (p<0,001), sexo (p=0,003), edad (p=0,006) y hipertensión arterial (p=0,013) con el Cog-DT. Los adultos mayores con mayor tiempo para realizar el Cog-DT fueron: los con fragilidad, con 0-4 años de estudio, del sexo femenino, con edad ≥80 años y con hipertensión arterial.

## Discusión

El objetivo del presente estudio fue analizar la influencia de la fragilidad y de la disminución cognitiva en el desempeño de la doble tarea en adultos mayores, considerando que la autonomía e independencia son esenciales en la vida de las personas.

La media de edad de los adultos mayores fue de 72,55 años y el intervalo etario más prevalente fue de adultos mayores (más jóvenes), cuyas edades variaron de 60 a 79 años. Ese intervalo etario es uno de las más preeminentes en Brasil^(4)^, el intervalo etario de 60 a 79 años representa 87,5% de los adultos mayores; en países de baja y media renta, se observa el mismo resultado. Además de eso, observamos mayor participación de mujeres en este estudio, lo que está de acuerdo con la literatura. El fenómeno resultante de la transición demográfica, en la cual el número de mujeres con 60 años o más se destaca del contingente masculino, es denominado de feminización de la edad^(18)^. En este estudio, la escolaridad media de la muestra fue de 5,15 años. Otros dos estudios encontraron en sus resultados una escolaridad media de 5,42 años y 5,0 años, respectivamente, lo que se asemeja a lo encontrado en este estudio. Es importante mencionar que la baja escolaridad está directamente relacionada a la fragilidad en adultos mayores^(3)^.

La prevalencia de la fragilidad en la población brasileña todavía no ha sido adecuadamente estimada, y los puntos de corte de los ítems que componen las escalas deben ser adaptados a los parámetros para esa población^(18)^. Este estudio identificó que 39,27% de los adultos mayores eran frágiles. De la misma forma, un estudio^(1)^ evaluó 360 adultos mayores con 65 años o más e identificó la prevalencia de fragilidad en 48,7% de la muestra estudiada. Además de eso, fueron identificados 48,7% de adultos mayores no frágiles y 32,2% de prefrágiles^(1)^. Es importante destacar que 59,2% de los adultos mayores de este estudio son prefrágiles, una condición que es reversible. Eso destaca la importancia de contar con estrategias orientadas a la prevención de la fragilidad en ambos sexos, referentes a la prevención de condiciones crónicas, siendo más importante en las mujeres^(19)^. Otro punto importante es el bajo porcentaje de adultos mayores no frágiles. Una posible explicación para ese resultado podría ser que los adultos mayores que participaron de la investigación eran individuos que buscaron los servicios de salud (lugar en que ocurrió la recolección de datos) presentando ya alguna queja de salud o diagnóstico de enfermedades, como hipertensión y artritis, presentes en esa muestra. Consecuentemente, esos individuos ya tenían algunas limitaciones de salud, que se reflejan en los cinco factores indicativos de la fragilidad. También destacamos que, por ejemplo, la prevalencia de la fragilidad en la población de individuos mayores varía mucho debido a la diversidad de escalas utilizadas, en los diferentes perfiles de adultos mayores evaluados y en los locales de estudio.

La velocidad de la marcha es un componente del constructo fragilidad^(2)^. El desempeño de la marcha es significativamente diferente en adultos mayores frágiles, con y sin disminución cognitiva, cuando comparado al grupo control; entre tanto, no fueron encontradas diferencias significativas entre esos grupos durante las pruebas de caminada habitual y doble tarea^(18)^. La ausencia de diferencias - en los parámetros de la marcha, entre los grupos frágiles con y sin disminución cognitiva, tanto en tareas simples como en tareas dobles - comprueba la estrecha relación entre esos dos trastornos y refuerza la idea de que, el comprometimiento cognitivo y la fragilidad comparten la misma etiología^(18)^.

En este estudio, la disminución cognitiva tuvo prevalencia de 57,48%, la que ocurrió de forma heterogénea e individual. Factores como edad, escolaridad y capacidad funcional pueden estar directamente relacionados a la prevalencia del comprometimiento cognitivo en adultos mayores^(8, 20)^.

A pesar de que, en este estudio no fue identificada una relación entre disminución cognitiva y disminución de la velocidad de marcha, los adultos mayores que obtuvieron mayores puntajes cognitivos fueron aquellos con mejor desempeño en la velocidad de marcha, en relación a las tareas dobles evaluadas. Se sabe que la cognición y marcha están íntimamente relacionadas^(5)^. Evidencias recientes demuestran que el comprometimiento cognitivo y las caídas también están relacionados; también, que el comprometimiento de la marcha y las caídas son más prevalentes en personas con demencia y que la prevalencia de comprometimientos en la marcha y caídas aumenta con la gravedad del comprometimiento cognitivo^(20)^.

La velocidad de marcha en la tarea doble no consigue distinguir las personas con cognición preservada de aquellas con comprometimiento cognitivo leve, a pesar de que permite diferenciar pacientes con la enfermedad de Alzheimer específicamente de otros perfiles cognitivos^(20)^. La estrecha relación entre cognición y marcha se debe a que comparten las estructuras cerebrales durante la ejecución simultánea de dos tareas. Las metodologías de la tarea doble suministran un ambiente que permite la manipulación de tareas motoras y cognitivas para evaluar la relación cognición-marcha^(21)^. Además de una tarea de procesamiento como resolución de problemas (por ejemplo, sustracción de 3 por 3), la atención y la demanda visual durante la caminada (como cargar un vaso en cuanto se camina) pueden perjudicar el desempeño de la marcha y el funcionamiento cognitivo, o los dos al mismo tiempo^(22)^.

Los hallazgos de este estudio muestran que la disminución cognitiva no fue relacionada al peor desempeño en la velocidad de marcha o en tareas dobles, tanto motoras como cognitivas. Sin embargo, fue constatado que cuanto mayor es el puntaje cognitivo de los adultos mayores, mejor será su desempeño en el tiempo de marcha evaluado.

Analizar la marcha como una tarea compleja es benéfico para la salud del adulto mayor, ya que puede aumentar su validad como marcador para identificación de la disminución cognitiva precoz^(23)^. La velocidad de la marcha puede ser utilizada como un índice de la función cognitiva y del estado de salud actual del individuo^(6)^.

El desarrollo de instrumentos - que evalúen, simultáneamente, déficits relacionados a la fragilidad física y cognitiva - es importante debido a la asociación entre el síndrome de la fragilidad y la cognición en adultos mayores. A pesar de eso, poco se sabe sobre de qué modo esos aspectos influencian el desempeño de pruebas de tarea doble^(23)^. Fue verificada la influencia del síndrome de la fragilidad y de la actividad física y cognición medidas en el TUG y TUG-DT; además de eso, los autores compararon los desempeños de ambas pruebas entre adultos mayores frágiles. Los resultados mostraron que no hubo diferencias en la cognición entre los grupos. Además de eso, cuando comparado al grupo frágil, el grupo no frágil exigió menos tiempo para realizar el TUG. En relación al TUG-DT, la cognición y la edad influenciaron el tiempo utilizado para realizar la tarea. Sin embargo, no fueron encontradas diferencias entre los grupos. Así, los adultos mayores frágiles tuvieron peor desempeño en el TUG cuando comparados a los participantes no frágiles; el test de doble tarea no diferenció adultos mayores frágiles de no frágiles, independientemente de su desempeño cognitivo^(23)^.

Las alteraciones relacionadas a la doble tarea en la velocidad de marcha son sensibles y diferencian personas saludables de aquellas con comprometimiento cognitivo. La velocidad de marcha es más lenta en personas con más edad y menos educadas^(6, 23)^. Ser mujer, soltero y tener baja renta son características del grupo de ritmo más lento; también, en ese grupo, la puntuación de la función cognitiva fue menor. En el mismo estudio, los autores también encontraron una correlación entre la velocidad de marcha más lenta y la disminución del función cognitiva y sugirieron que la rehabilitación geriátrica debe incluir no apenas el entrenamiento de marcha y el fortalecimiento de los músculos de las pernas, pero también el entrenamiento de las habilidades cognitivas frontales y generales para mejor desempeño de la marcha^(6, 22, 23)^.

La realización de tareas secundarias durante la caminada disminuyó la velocidad de marcha, lo que sugiere que la marcha depende del funcionamiento cognitivo^(5, 9)^. Sin embargo, también es posible que la disminución de la velocidad de marcha sea debida al estado de fragilidad de la población de adultos mayores^(6)^.

La relación entre cognición y marcha durante la caminada de doble tarea puede ser una herramienta para monitorear la deterioración de la marcha a lo largo del envejecimiento, inclusive las leves. Es claro que un desafío cognitivo durante la ejecución de una tarea motora interfiere en su desempeño. En general, la capacidad de lidiar con una tarea doble disminuye progresivamente con el envejecimiento, a pesar de que las razones permanezcan poco claras^(9)^. El presente estudio mostró que la presencia de hipertensión arterial influenció el empeoramiento del desempeño de la marcha de los participantes. Por otro lado, la velocidad de marcha es considerada un predictor precoz de la enfermedad cardiovascular^(6)^. Un estudio realizado en Taiwán investigó la asociación entre la velocidad de la caminada y el riesgo de enfermedad cardiovascular entre poblaciones de media-edad y adultos mayores, en la comunidad. Su conclusión sugiere que la velocidad lenta de la marcha fue asociada a un riesgo aumentado de enfermedad cardiovascular, en personas de media-edad, pero no en adultos mayores^(6)^.

En ese sentido, los resultados de este estudio son corroborados con los hallazgos de un estudio recientemente realizado^(6)^, en el cual el desempeño de la velocidad de marcha fue peor en adultos mayores con edad más avanzada y menor escolaridad, con menores puntajes cognitivos y con hipertensión arterial.

Este estudio presenta algunas limitaciones. Existen diferencias en la estandarización de la evaluación de la marcha con la evaluación cognitiva, descritas en la literatura. En relación a la marcha, los estudios que evalúan la marcha de adultos mayores por medio del TUG no siguen la distancia específica como la utilizada en el presente estudio. Eso dificulta la comparación de estudios y el análisis de poblaciones. Lo mismo sucede con la evaluación cognitiva, existen varios instrumentos para evaluar esa variable y es difícil comparar el desempeño con diferentes medidas. A pesar de esas limitaciones, los estudios deben ser incentivados a utilizar diferentes instrumentos de evaluación, si es necesario, para ofrecer confiabilidad y reproductibilidad para las evaluaciones en Unidades de Salud.

El objetivo de detectar déficits precozmente es fundamental para identificar necesidades, organizar, implementar y reevaluar la eficiencia de las intervenciones, con la finalidad de promover mejorías en la calidad de vida de los adultos mayores; este objetivo también integra las habilidades y competencias de los profesionales de la salud. De esa forma, ese conocimiento contribuirá para mejorar la implementación de intervenciones orientadas a mejorar la marcha y/o mantener la cognición.

## Conclusión

Con base en el objetivo propuesto - por medio del análisis de la influencia de la fragilidad y de la disminución cognitiva, en el desempeño de doble tarea en adultos mayores - este estudio demostró que la fragilidad es un factor con mayor interferencia en el desempeño de la doble tarea, en adultos mayores, cuando comparado al desempeño cognitivo. Es importante mencionar que la fuerte asociación entre marcha y cognición ha sido descrita en muchos estudios. También, vale destacar que adultos mayores con déficits de marcha tienen mayor riesgo de desarrollar déficits cognitivos que están asociados al empeoramiento de la marcha.
